# Cardiorespiratory Fitness, Multimorbidity Risk, and 15-Year Trajectories in Chronic Disease Accumulation: A Prospective Longitudinal Study

**DOI:** 10.1016/j.jacadv.2025.102198

**Published:** 2025-10-08

**Authors:** Liyao Xu, Shuqi Wang, Maiwulamujiang Maimaitiyiming, Wenzhe Yang, Sakura Sakakibara, Xiuying Qi, Yaogang Wang, Abigail Dove

**Affiliations:** a School of Public Health, Tianjin Medical University, Tianjin, China; b Key Laboratory of Prevention and Control of Major Diseases in the Population (Tianjin Medical University), Ministry of Education, Tianjin, China; c Aging Research Center, Department of Neurobiology, Care Sciences and Society, Karolinska Institutet, Stockholm, Sweden

**Keywords:** cardiorespiratory fitness, chronic disease, disease trajectories, multimorbidity, UK Biobank

## Abstract

**Background:**

Cardiorespiratory fitness (CRF) has been linked to lower risk of individual chronic diseases, but little is known about the CRF in relation to multimorbidity.

**Objectives:**

The authors investigated the association between CRF and multimorbidity risk and explored differences in the trajectories of chronic disease accumulation at varying levels of CRF.

**Methods:**

The study included 38,348 adults from the UK Biobank (mean age 55.21 ± 8.15 years; 51.95% female) who were followed for up to 15 years to detect the incidence of 59 common chronic diseases. CRF was estimated using a 6-minute submaximal exercise test and tertiled as low, moderate, and high (after standardization by age and sex). Multimorbidity was defined as the presence of 2 or more chronic diseases. Data were analyzed using Cox regression, Laplace regression, and linear mixed-effects models.

**Results:**

During the follow-up (median [IQR]: 11.57 [7.39-11.76] years), 15,368 (40.08%) participants developed multimorbidity. The risk of multimorbidity was 21% lower in participants with high compared to low CRF (HR: 0.79 [95% CI: 0.76-0.83]). The median time to multimorbidity onset was 1.27 (95% CI: 1.01-1.54) years later for those with high compared to low CRF. Moreover, participants with high CRF experienced a significantly slower annual rate of chronic disease accumulation (β = −0.043 [−0.050 to −0.036]).

**Conclusions:**

High CRF is associated with lower multimorbidity risk, delayed onset of multimorbidity, and significantly slower accumulation of chronic diseases. The findings highlight the importance of CRF for healthy longevity.

The past decades have seen an unprecedented increase in global average life expectancy.^1^^,^^2^ While longer life expectancy is a victory for public health, population aging raises significant challenges—chief among them a rise in the prevalence of multimorbidity, that is, the coexistence of 2 or more chronic diseases in the same individual.^3^^,^^4^ An estimated one-third of adults worldwide are affected by multimorbidity,^5^^,^^6^ and this increases sharply with age from 30% among individuals aged 45 to 64 years to 65% among those aged 65 to 84 years to 82% among those above the age of 85 years.^7^^,^^8^ Multimorbidity contributes to higher health care expenditures, increased disability, and a greater risk of mortality.^9^, ^10^, ^11^ Therefore, strategies are needed to prevent or delay the onset of multimorbidity.

Cardiorespiratory fitness (CRF) refers to the body’s ability to efficiently deliver oxygen to muscles during physical activity and is typically measured in metabolic equivalents (METs).^12^ Higher CRF has a well-established relationship with lower all-cause mortality and reduced risk of specific chronic diseases like diabetes and cardiovascular disease.^12^, ^13^, ^14^, ^15^, ^16^ Specifically, in meta-analyses, each 1-MET improvement in CRF has been related to an 8% lower risk of type 2 diabetes,^17^ 13% lower risk of cardiovascular disease, and 15% lower risk of all-cause mortality.^12^ Along similar lines, higher CRF has further been related to lower risk of the development of comorbid type 2 diabetes and cardiovascular disease.^12^ However, evidence is lacking on how CRF affects the overall accumulation chronic diseases or the development of multimorbidity.

The present study comprehensively investigates the relationship between CRF and multimorbidity, leveraging 15-year longitudinal data from >38,000 middle-aged and older adults from the UK Biobank. Specifically, we aimed to: 1) examine whether and to what extent high CRF can reduce the risk and delay the onset time of multimorbidity; and 2) map long-term trajectories in the accumulation of different chronic diseases at different levels of CRF.

## Methods

### Study design and population

The UK Biobank is a prospective longitudinal study including >500,000 UK adults between the ages of 40 and 69. The baseline examination took place between 2006 and 2010 at 1 of 22 assessment centers across the country and consisted of clinical and physical assessments and completion of a series of questionnaires on sociodemographic and lifestyle factors. Changes in health status were monitored via linkage with medical records for a maximum of 15 years (until January 2022).

Selection of the study population is illustrated in Supplemental Figure 1. The analysis was first restricted to 61,887 participants with available data on baseline CRF. We then excluded 23,495 participants with any prevalent chronic disease at baseline and 85 with outlier values for CRF (ie, ≥3 SDs from the mean). This left a study sample of 38,348 chronic disease-free participants for the analysis of the association between CRF and multimorbidity risk and trajectory.

Data collection procedures have been approved by the National Health Services’ National Research Ethics Service (Ref 11/NW/0382), and the use of the data for the present analyses was additionally approved by the Regional Ethical Review Board in Stockholm (2024-00520-01). All participants provided written informed consent at baseline.

### Assessment of CRF

CRF was measured at baseline using a 6-minute submaximal exercise test on a stationary bike (eBike Comfort Ergometer, General Electric, firmware version 1.7) while wearing a 4-lead electrocardiographic monitor.^18^ Participants’ risk category was first assessed through interviews to ensure that it was safe to perform this activity. Then each individual’s maximum workload was calculated according to their age, height, weight, resting heart rate, and sex. All participants were asked to cycle at 60 revolutions per minute throughout the duration of the submaximal exercise test, and protocols were individualized according to participants’ risk category: 1) minimal risk (cycling at 50% of the predicted maximum workload); 2) low risk (cycling at 35% of the predicted maximum workload); and 3) medium risk (cycling at a constant level of 30 W for women and 40 W for men). A detailed description of the CRF protocol has been published elsewhere^19^ and is illustrated in the flowchart in Supplemental Figure 2. More information can also be found at https://biobank.ndph.ox.ac.uk/showcase/refer.cgi?id=100229.

Participants’ heart rate and workload were continuously monitored during the cycle test to assess their CRF levels. A linear regression analysis was conducted to estimate the regression line for the relationship between each participant’s heart rate and workload. The predicted maximum heart rate (208-0.7 × age) was then substituted into the formula to calculate the maximal work rate.^20^ Finally, CRF was ascertained by first calculating maximum oxygen consumption (in mL kg^−1^ min^−1^) using the equation 7 + (10.8 × maximal work rate)/body weight (kg) and converting into METs, where 1 MET = 3.5 mL kg^−1^ min^−1^.^21^ CRF values were standardized based on age (grouped by decade) and sex and tertiled into a low, moderate, and high group.

### Assessment of multimorbidity

A total of 59 common chronic diseases were identified through medical records according to the 10th edition of the International Classification of Diseases (Supplemental Table 1).^3^ Multimorbidity was defined as the coexistence of 2 or more of these conditions. Once a chronic disease was diagnosed, it was considered present for the duration of the follow-up.

Chronic diseases were further classified into 3 subcategories: 1) *metabolic diseases* (including hypertension, diabetes, dyslipidemia, obesity, and other metabolic diseases)^22^^,^^23^; 2) *cardiovascular diseases* (including ischemic heart disease, heart failure, atrial fibrillation, cerebrovascular disease, cardiac valve diseases, bradycardias or conduction disorders, peripheral vascular disease, and other cardiovascular diseases)^24^; and 3) *neuropsychiatric diseases* (including depression and mood disorders, dementia, neurotic or stress-related and somatoform diseases, migraine and facial pain syndromes, peripheral neuropathy, Parkinson disease or parkinsonism, epilepsy, schizophrenia and delusional diseases, multiple sclerosis, other psychiatric or behavioral diseases, and other neurological diseases).^24^

### Assessment of covariates

Information on sociodemographic characteristics (age, sex, education, socioeconomic status, and race) and lifestyle factors (smoking status, alcohol consumption, and physical activity) was collected at baseline through computerized touch screen questionnaires (Supplemental Table 2). Education level was dichotomized according to whether participants had completed college/university. Socioeconomic status was measured using the Townsend Deprivation Index, a measure of neighborhood-level socioeconomic deprivation based on unemployment, household overcrowding, and car/home ownership in a given postcode of residence.^25^ Race was self-reported according to the 2001 UK census categories and dichotomized as White or non-White (including Asian, Black, multiracial, or other). Smoking status and alcohol consumption were self-reported and categorized as never, previous, or current. Physical activity was measured using the International Physical Activity Questionnaire, which contains items about the frequency and duration of light, moderate-intensity, and vigorous-intensity activities over the past 7 days. Physical activity was classified as low (<600 MET/week), moderate (600-3000 MET/week), or high (≥3000 MET/week).^26^

### Statistical analysis

Baseline characteristics of the study population by CRF category were assessed using chi-square tests for categorical variables, and one-way analysis of variance tests or Kruskal-Wallis tests for continuous variables.

A Sankey plot was used to visualize the longitudinal accumulation of chronic diseases among the study participants, illustrating the number of individuals with high, moderate, and low CRF at baseline that developed 0, 1, 2, or ≥3 chronic diseases by the end of follow-up. Cox regression models were used to evaluate HRs and 95% CIs for the association between CRF status and the development of multimorbidity. Follow-up was measured as the time (in years) from study entry to the onset of multimorbidity (ie, diagnosis of a second chronic disease), death, or last available follow-up (January 20, 2022), whichever came first. The proportional hazard assumption was assessed using Schoenfeld residuals regressed against follow-up time; no violations were observed. Laplace regression models were used to estimate the 50th percentile differences in the median time (in years) to the development of multimorbidity as a function of CRF level.^27^ Given possible differences in prevalence and etiology of multimorbidity at various ages, we conducted age-stratified analyses (middle-aged [<60 years] vs older [≥60 years]). Multiplicative interactions between CRF and age were tested by incorporating the CRF × age group cross-product term into the models. Next, linear mixed-effects models were used to estimate standardized β-coefficients and 95% CIs for the longitudinal association between CRF status and change in the number of comorbid chronic diseases (from baseline until death or the end of follow-up). All analyses were adjusted for age, race, education, socioeconomic status, smoking status, alcohol consumption, and physical activity.

In sensitivity analysis, we repeated the analyses after 1) using nonage/sex-standardized CRF values to create the CRF groups (to provide unstandardized regression coefficients to facilitate comparison with other studies)^28^; 2) excluding participants who were diagnosed with multimorbidity within the first 3 years of follow-up (to reduce the possibility of reverse causality); 3) using Fine-Gray subdistribution hazard models with death as a competing risk; and 4) imputing missing values for covariates using multiple imputation by chained equations.

*P* values <0.05 were considered statistically significant. Analyses were performed using Stata SE 15.0 (StataCorp).

## Results

### Baseline characteristics of the study population

The mean age of the study sample at baseline was 55.21 ± 8.15 years, and 51.95% were female. Mean CRF was −1.02 ± 0.40 MET in the low CRF group, −0.10 ± 0.23 MET in the moderate CRF group, and 1.12 ± 0.70 MET in the high CRF group. Participants with low CRF were more likely to be older, non-White, have a lower education level and socioeconomic status, be physically inactive, and abstain from smoking and alcohol drinking compared to their counterparts with moderate or high CRF (Table 1).

**Table 1:** Baseline Characteristics of the Study Population by Cardiorespiratory Fitness Level (N = 38,348)

	CRF			*P* Value
	Low (n = 12,785)	Moderate (n = 12,782)	High (n = 12,781)	
CRF, MET	−1.02 ± 0.40	−0.10 ± 0.23	1.12 ± 0.70	<0.001
Age, y	55.47 ± 8.26	55.23 ± 8.16	54.92 ± 8.01	0.003
Sex				1.000
Female	6,642 (51.95)	6,640 (51.95)	6,639 (51.94)	
Male	6,143 (48.05)	6,142 (48.05)	6,142 (48.06)	
College/university-educated	4,415 (34.98)	5,065 (40.05)	6,110 (48.09)	<0.001
White	10,943 (86.19)	11,048 (87.05)	11,202 (88.12)	<0.001
Townsend Deprivation Index	−1.74 (−3.04, 0.99)	−1.97 (−3.54, 0.48)	−1.95 (−3.57, 0.54)	<0.001
Smoking status				<0.001
Never	7,511 (59.07)	7,350 (57.77)	7,125 (55.97)	
Previous	4,051 (31.86)	4,219 (33.16)	4,408 (34.62)	
Current	1,154 (9.08)	1,153 (9.06)	1,198 (9.41)	
Alcohol consumption				<0.001
Never	589 (4.62)	470 (3.69)	311 (2.44)	
Previous	372 (2.92)	325 (2.55)	270 (2.12)	
Current	11,786 (92.46)	11,954 (93.76)	12,176 (95.45)	
Physical activity				<0.001
Low	2,196 (20.90)	1,741 (16.16)	1,218 (10.88)	
Moderate	5,446 (51.84)	5,755 (53.41)	5,829 (52.08)	
High	2,864 (27.26)	3,280 (30.44)	4,146 (37.04)	

MET = metabolic equivalents; CRF = cardiorespiratory fitness.

Values are mean ± SD, n (%), or median (IQR).

Missing data: 375 for education, 248 for race, 46 for Townsend Deprivation Index, 179 for smoking status, 95 for alcohol consumption, and 5,873 for physical activity.

### CRF and risk of multimorbidity

**Figure 1:**
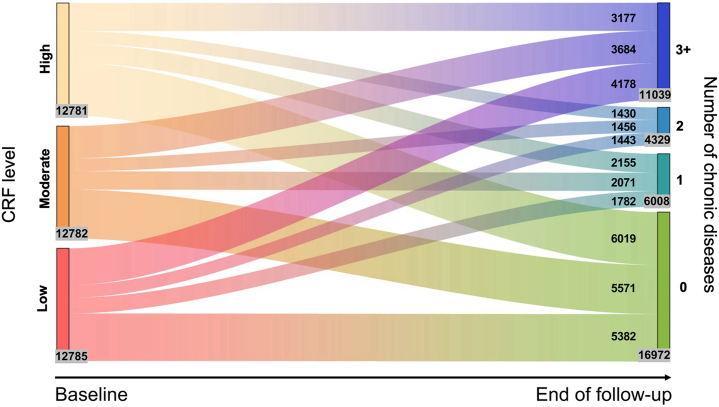
**Evolution in Chronic Disease Status by CRF Level** The height of the boxes and the thickness of the stripes are proportional to the number of participants in each state. CRF = cardiorespiratory fitness.

Over the follow-up period (median [IQR]: 11.57 [7.39-11.76] years), 6,008 (15.67%) participants developed one chronic disease, 4,329 (11.29%) developed 2 chronic diseases, and 11,039 (28.79%) developed 3 or more chronic diseases. Figure 1 presents a Sankey plot illustrating the longitudinal accumulation of chronic diseases by baseline CRF category. Participants with low CRF were more likely to progress to multimorbidity: by the end of follow-up, 4,178 (33%) individuals from the low CRF group had developed 3 or more chronic conditions, compared with 3,684 (29%) from the moderate CRF group and 3,177 (25%) from the high CRF group. Consistent with this, in multiadjusted Cox regression, higher CRF (as a continuous variable; per 1-SD increment) was dose-dependently related to lower risk of multimorbidity (HR: 0.91[0.89-0.93]). Compared to participants in the low CRF group, the risk of multimorbidity was 10% and 21% lower among participants with moderate CRF (HR: 0.90 [95% CI: 0.87-0.94]) and high CRF (HR: 0.79 [95% CI: 0.76-0.83]) (Table 2). Furthermore, multiadjusted Laplace regression showed that the development of multimorbidity was delayed by 0.54 and 1.27 years among participants with moderate and high CRF, respectively (Table 3).

**Table 2:** HRs and 95% CIs for the Association Between Cardiorespiratory Fitness and Multimorbidity Risk: Results From Cox Regression

CRF	N	Multimorbidity Risk		
		No. of Cases	HR (95% CI)	*P* Value
Continuous (per 1-SD increment)	38,348	15,368	0.91 (0.89-0.93)	<0.001
Categorical				
Low	12,785	5,621	1.00 (reference)	-
Moderate	12,782	5,140	0.90 (0.87-0.94)	<0.001
High	12,781	4,607	0.79 (0.76-0.83)	<0.001

Multimorbidity is defined as the presence of at least 2 chronic diseases.

Abbreviation as in Table 1.

Models were adjusted for age, race, education, socioeconomic status, smoking status, alcohol consumption, and physical activity.

**Table 3:** Mean Time (in Years) to the Onset of Multimorbidity According to Cardiorespiratory Fitness Level: Results From Laplace Regression

CRF	N	Multimorbidity Onset		
		No. of Cases	50th PD (95% CI)	*P* Value
Continuous (per 1-SD increment)	38,348	15,368	0.52 (0.41-0.63)	<0.001
Categorical				
Low	12,785	5,621	0.00 (reference)	-
Moderate	12,782	5,140	0.54 (0.29-0.78)	<0.001
High	12,781	4,607	1.27 (1.01-1.54)	<0.001

Multimorbidity is defined as the presence of at least 2 chronic diseases.

PD = percentile difference; other abbreviation as in Table 1.

Models were adjusted for age, race, education, socioeconomic status, smoking status, alcohol consumption, and physical activity.

**Figure 2:**
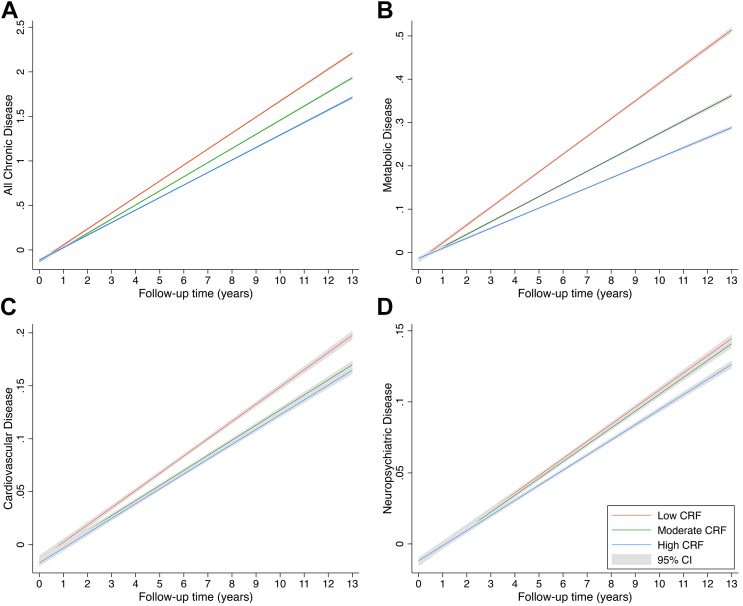
**15-Year Trajectories in Chronic Disease Accumulation by CRF Level** Graphs depict trajectories in the accumulation of (A) all chronic diseases, (B) metabolic disease, (C) cardiovascular disease, and (D) neuropsychiatric disease, as a function of CRF level. Models were adjusted for age, race, education, socioeconomic status, smoking status, alcohol consumption, and physical activity. Abbreviation as in Figure 1.

After stratification by age, higher CRF remained associated with lower risk of multimorbidity in both middle-aged (HR: 0.89 [95% CI: 0.86-0.91]) and older (HR: 0.94 [95% CI: 0.91-0.96]) participants, although the magnitude of the association was larger among middle-aged group (Figure 2). We detected a significant multiplicative interaction between CRF (high vs low) and age group with respect to multimorbidity risk (*P* = 0.018).

### CRF and trajectories in chronic disease accumulation

**Figure 3:**
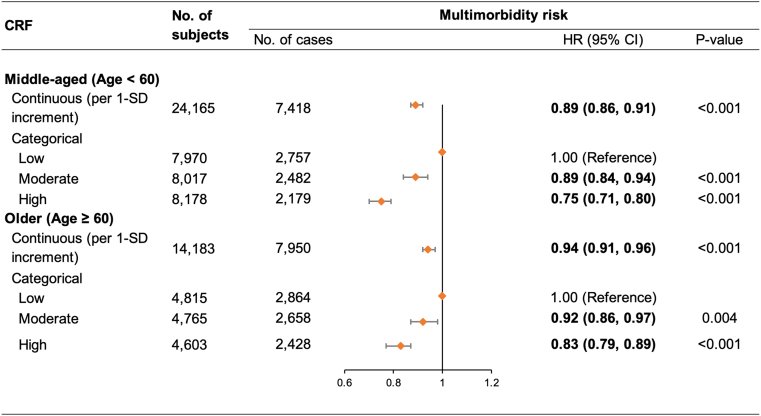
**Age-Stratified Analyses for the Association Between CRF and Multimorbidity** Models were adjusted for race, education, socioeconomic status, smoking status, alcohol consumption, and physical activity. HR (95% CI) and *P* value of multiplicative interaction for multimorbidity risk: CRF (moderate vs low) and age: 0.96 (0.89-1.05), *P* = 0.387, false discovery rate-adjusted q = 0.387; CRF (high vs low) and age: 0.89 (0.82-0.97), *P* = 0.009, false discovery rate-adjusted q = 0.018. Abbreviation as in Figure 1.

In linear mixed-effects models, accumulation of chronic diseases over the 15-year follow-up was significantly slower among individuals with moderate (β = −0.021 [−0.028 to −0.015]) or high (β = −0.043 [−0.050 to −0.036]) CRF compared to low CRF. Specifically, participants with high CRF compared to low CRF had significantly slower accumulation of metabolic diseases (β = −0.019 [−0.021 to −0.017]), cardiovascular diseases (β = −0.003 [−0.004 to −0.001]), and neuropsychiatric diseases (β = −0.002 [−0.003 to −0.001]). Similarly, moderate CRF was linked with significantly slower accumulation of metabolic diseases (β = −0.012 [−0.014 to −0.010]) and cardiovascular diseases (β = −0.002 [−0.004 to −0.001]) (Figure 3; Table 4).

**Table 4:** Standardized β-Coefficients and 95% CIs for the Association Between Cardiorespiratory Fitness and Number of Chronic Diseases Accumulated Over the 15-Year Follow-Up

CRF	All Chronic Disease		Metabolic Disease		Cardiovascular Disease		Neuropsychiatric Disease	
	β (95% CI)	*P* Value	β (95% CI)	*P* Value	β (95% CI)	*P* Value	β (95% CI)	*P* Value
Continuous (per 1-SD increment) × time	−0.018 (−0.020 to −0.015)	<0.001	−0.008 (−0.009 to −0.007)	<0.001	−0.001 (−0.002 to −0.001)	<0.001	−0.001 (−0.001 to −0.000)	<0.001
Categorical × time								
Low	0.000 (reference)	-	0.000 (reference)	-	0.000 (reference)	-	0.000 (reference)	-
Moderate	−0.021 (−0.028 to −0.015)	<0.001	−0.012 (−0.014 to −0.010)	<0.001	−0.002 (−0.004 to −0.001)	0.007	−0.000 (−0.001 to 0.001)	0.799
High	−0.043 (−0.050 to −0.036)	<0.001	−0.019 (−0.021 to −0.017)	<0.001	−0.003 (−0.004 to −0.001)	<0.001	−0.002 (−0.003 to −0.001)	0.002

Abbreviation as in Table 1.

Models were adjusted for age, race, education, socioeconomic status, smoking status, alcohol consumption, and physical activity.

### Sensitivity analysis

Results remained consistent when we repeated the analyses after: 1) utilizing non-age/sex-standardized CRF values (Supplemental Tables 3 and 4); 2) excluding participants who were diagnosed with multimorbidity within the first 3 years of follow-up (Supplemental Tables 5 and 6); 3) evaluating the competing risk of death using Fine-Gray subdistribution hazard models (Supplemental Table 7); and 4) using multiple imputation to handle missing values for covariates (Supplemental Tables 8 to 10).

## Discussion

In this large prospective longitudinal study of >38,000 participants, we found that moderate to high CRF levels were associated with a lower risk of multimorbidity, a >1 year delay in the onset of multimorbidity, and slower accumulation of chronic diseases (including metabolic, cardiovascular, and neuropsychiatric diseases) over time (Central Illustration).

**Central Illustration:**
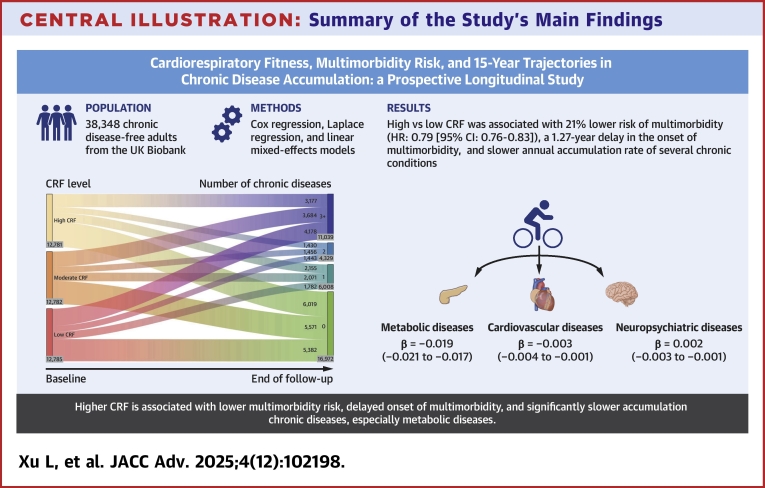
**Summary of the Study’s Main Findings** Abbreviation as in Figure 1.

The present study contributes valuable insight into role of CRF in the development of multimorbidity and trajectories in the accumulation of multiple, comorbid chronic diseases. To date, CRF has been linked to increased risk of comorbid type 2 diabetes and cardiovascular disease,^16^ but evidence on the relationship between CRF and overall chronic disease burden is limited—though a growing literature has explored CRF in relation to individual chronic diseases. For instance, a recent meta-analysis of 34 cohort studies reported that higher CRF was associated with lower all-cause mortality and lower risk of mortality due to cardiovascular disease and cancer.^29^ Additional studies have linked high CRF to lower risk of hypertension,^30^ dementia,^31^ and sleep apnea^32^ and identified low CRF as a risk factor for depression and other common neuropsychiatric disorders.^33^^,^^34^

The strong associations uncovered in this study between high CRF and lower risk of multimorbidity are consistent with the notion that CRF may be a proxy for overall objective health status, as it reflects the collective function of several major organ systems, namely respiratory, cardiovascular, and skeletal muscle.^35^ That said, CRF may exert a stronger influence on certain chronic diseases or disease clusters than others. Interestingly, in our analysis of trajectories in the accumulation of different clusters of chronic diseases, moderate and high CRF were most strongly related to a slowing in the accumulation of metabolic diseases like obesity, hypertension, diabetes, and dyslipidemia. Slightly weaker associations were found between moderate and high CRF and the accumulation of cardiovascular diseases (like ischemic heart disease, heart failure, atrial fibrillation, and stroke), and finally neuropsychiatric diseases (like depression, dementia, or Parkinson disease), where the association remained only for participants with high CRF. This could reflect the complex interrelationships between different chronic diseases, where metabolic diseases may increase the risk of cardiovascular diseases and both metabolic and cardiovascular diseases may increase the risk of neuropsychiatric conditions.

Notably, we found evidence of age-heterogeneity in the relationship between CRF and multimorbidity, whereby the apparent protective effect of high CRF on multimorbidity risk was more pronounced in middle-aged adults (<60 years) compared to older adults (≥60 years). This finding is consistent with previous studies showing accelerated development of multimorbidity at older ages.^36^^,^^37^ In addition to accelerating the accumulation of chronic diseases, aging could also dampen the protective effect of high CRF through mechanisms such as cellular senescence, immunosenescence, and increased inflammation.^37^ From a public health perspective, this finding suggests that middle-aged adults may benefit more from potential interventions aimed at improving CRF to reduce the risk of multimorbidity. This warrants deeper consideration in future studies.

Further research is also needed to better understand the biological mechanisms connecting CRF and lower risk of multimorbidity. Previous studies have linked high CRF to lower levels of chronic inflammation,^38^ enhanced immune function,^39^ lower levels of circulating steroid hormones,^40^ reduced accumulation of visceral adipose tissue,^41^ and increased antioxidant capacity^42^—all of which may offer protection against the development of a range of chronic diseases. Moreover, a recent systematic review of metabolomic studies on CRF reported that CRF is related to metabolic signatures related to circulation and cardiac metabolism, including higher levels of metabolites such as circulating lyso-acylglycerophosphocholines, muscle acylcarnitine, and cholesterol esters, and lower levels of metabolites such as ceramides, acylcarnitine, and circulating glycerol.^43^

### Strengths and limitations

A major strength of this study lies in the comprehensive measurement of CRF in a large population of >38,000 people. The use of a submaximal (rather than maximal) exercise test enabled greater participation in the CRF test, especially among older adults and those with poorer health status. The study additionally benefits from a long 15-year follow-up, during which the onset of 59 common chronic diseases was monitored. The large sample size enabled us to specifically consider specific disease clusters that are especially relevant for the aging population, including cardiovascular, metabolic, and neuropsychiatric diseases. Nonetheless, some limitations should be acknowledged. First, our study is subject to selection bias insofar as the UK Biobank study population is healthier and more socioeconomically advantaged than the general UK population,^44^ and eligibility for the submaximal exercise test excluded individuals with certain health conditions (ie, high weight, high blood pressure, chest pain at rest, pacemaker, etc.). Therefore, it is plausible that our study sample had a lower risk of chronic diseases and lower variability in CRF compared to the general population, limiting the generalizability of our findings and potentially contributing to an underestimation of the association between CRF and multimorbidity. Second, although estimating CRF from submaximal exercise tests is a practical approach for large-scale studies, this method may lead to an overestimation of VO_2max_. In previous studies, estimated VO_2max_ from submaximal exercise tests showed moderate to high correlation with the gold standard measurement of directly measured VO_2max_, with Pearson’s r ranging from 0.68 to 0.74.^45^ Third, incident chronic diseases were primarily ascertained through medical records and were therefore likely to be underdiagnosed in this study. Relatedly, reverse causality is possible insofar as the presence of a prodromal or as-of-yet undiagnosed chronic disease may have contributed to poorer CRF at baseline, potentially contributing to an exaggeration of the association between high CRF and lower chronic disease risk. However, we accounted for this in sensitivity analyses excluding participants who developed chronic diseases within the first 3 years of follow-up, and the results remained consistent (Supplemental Tables 5 and 6). Finally, even after adjusting for many confounding factors, we cannot rule out the possibility of residual confounding by unmeasured confounders (ie, diet, medication adherence, and genetic predisposition). Future studies are warranted to further validate the causal relationships observed in this study.

## Conclusions

Our study provides evidence that higher CRF is associated with lower multimorbidity risk, delayed onset of multimorbidity, and significantly slower accumulation chronic diseases, especially metabolic diseases. Together, these findings highlight the maintenance of CRF as a potential strategy to prevent or delay the development of multimorbidity as individuals age.

Perspectives **COMPETENCY IN MEDICAL KNOWLEDGE:** Having higher CRF was associated with significantly lower risk of multimorbidity and significantly slower accumulation of chronic diseases over 15 years of follow-up.

**TRANSLATIONAL OUTLOOK:** Future studies should explore whether interventions to improve CRF can prevent the development of chronic diseases and multimorbidity.

## Funding support and author disclosures

This study was supported by grants from 10.13039/501100008599Alzheimerfonden, 10.13039/100019796Demensfonden, and the 10.13039/100010771Loo and Hans Osterman Foundation for Medical Research. The funders had no role in the design and conduct of the study, the collection, management, analysis, and interpretation of the data, the preparation, review, or approval of the manuscript, or the decision to submit the manuscript for publication. The authors have reported that they have no relationships relevant to the contents of this paper to disclose.
