# Supplementary material for: Cardiorespiratory Fitness, Multimorbidity Risk, and 15-Year Trajectories in Chronic Disease Accumulation: A Prospective Longitudinal Study
Source: JACC Adv. 2025 Oct 8;4(12):102198. doi: 10.1016/j.jacadv.2025.102198 (PMC12805149; doi:10.1016/j.jacadv.2025.102198)
Supplement: Supplemental Material [file mmc1.docx]

**Supplemental Figure 1.** Flowchart of the study population.

Excluded 23,539 participants:

- With one or more chronic disease at baseline (n=23,495)
- With outlier values (≥3 standard deviations from the mean) for cardiorespiratory fitness (n=85)

Participants with baseline data on cardiorespiratory fitness (n=61,887)

Analytical sample (n=38,348)

**Supplemental Figure 2.** Flowchart for deriving cardiorespiratory fitness using ECG data collected from sub-maximal bike tests.

Excluded 9,614 who were ineligible for ECG testing or fell into the “high risk” category.

Excluded 6 who did not perform the bike test.

Sub-maximal bike tests offered and consent received at baseline (N=79,186)

Performed bike test (N=69,566)

Excluded 6,169 who did not complete the test.

Excluded 1,396 with invalid data (missing or 0) for heart rate (n=260), workload (n=1,145), or cadence (n=1,129).

Excluded 2 with missing data on resting heart rate.

Data obtained from bike tests (N=61,999)

Excluded 105 with abnormal observations, including:

- Test heart rate than resting heart rate (n=19).
- No valid data for cadence within the range of 35-125 RPM (n=49).
- No valid data for satisfying both cadence and heart rate (n=37).

Data available for calculate maximal work rate (N=61,894)

Excluded 6 with no valid data from the 2-min constant phase of the bike test.*

Excluded 1 with missing data on baseline weight.

Data available for calculation of cardiorespiratory fitness level (N=61,887)

* 402 participants had a negative β-coefficient for the relationship between workload and heart rate (i.e., biologically implausible), and their maximal workload value was instead derived using ECG data from the 2-min constant phase plus resting pulse rate. Because 6 of these 402 individuals did not have valid data from the 2-minute constant phase, they were excluded from the CRF calculation.

Abbreviations: ECG, electrocardiographic; RPM, revolutions per minute.

**Supplemental Table 1.** International Classification of Diseases (ICD) 10 codes used as inclusion and exclusion criteria to define the 59 chronic diseases.

| **ALLERGY** | |
| --- | --- |
| **Included ICD-10 codes and labels** | |
| J301 | Allergic rhinitis due to pollen |
| J302 | Other seasonal allergic rhinitis |
| J303 | Other allergic rhinitis |
| J304 | Allergic rhinitis, unspecified |
| J450 | Predominantly allergic asthma |
| K522 | Allergic and dietetic gastroenteritis and colitis |
| L20 | Atopic dermatitis |
| L23 | Allergic contact dermatitis |
| L500 | Allergic urticaria |
| Z516 | Desensitization to allergens |
| **ANEMIA** | |
| **Included ICD-10 codes and labels** | |
| D50 | Iron deficiency anaemia |
| D51 | Vitamin B12 deficiency anaemia |
| D52 | Folate deficiency anaemia |
| D53 | Other nutritional anaemias |
| D55 | Anaemia due to enzyme disorders |
| D56 | Thalassaemia |
| D57 | Sickle-cell disorders |
| D58 | Other hereditary haemolytic anaemias |
| D59 | Acquired haemolytic anaemia |
| D60 | Acquired pure red cell aplasia [erythroblastopenia] |
| D61 | Other aplastic anaemias |
| D63 | Anaemia in chronic diseases classified elsewhere |
| D64 | Other anaemias |
| **Excluded ICD-10 codes and labels** | |
| D563 | Thalassaemia trait |
| D590 | Drug-induced autoimmune haemolytic anaemia |
| D592 | Drug-induced nonautoimmune haemolytic anaemia |
| D593 | Haemolytic-uraemic syndrome |
| D596 | Haemoglobinuria due to haemolysis from other external causes |
| D601 | Transient acquired pure red cell aplasia |
| D611 | Drug-induced aplastic anaemia |
| D612 | Aplastic anaemia due to other external agents |
| D642 | Secondary sideroblastic anaemia due to drugs and toxins |
| **ASTHMA** | |
| **Included ICD-10 codes and labels** | |
| J45 | Asthma |
| **ATRIAL FIBRILLATION** | |
| **Included ICD-10 codes and labels** | |
| I48 | Atrial fibrillation and flutter |
| **AUTOIMMUNE DISEASES** | |
| **Included ICD-10 codes and labels** | |
| I731 | Thromboangiitis obliterans |
| L10 | Pemphigus |
| L12 | Pemphigoid |
| L40 | Psoriasis |
| L41 | Parapsoriasis |
| L93 | Lupus erythematosus |
| L94 | Other localized connective tissue disorders |
| L95 | Vasculitis limited to skin, not elsewhere classified |
| M30 | Polyarteritis nodosa and related conditions |
| M31 | Other necrotizing vasculopathies |
| M32 | Systemic lupus erythematosus |
| M33 | Dermatopolymyositis |
| M34 | Systemic sclerosis |
| M35 | Other systemic involvement of connective tissue |
| M36 | Systemic disorders of connective tissue in diseases classified elsewhere |
| **Excluded ICD-10 codes and labels** | |
| L105 | Drug-induced pemphigus |
| M320 | Drug-induced systemic lupus erythematosus |
| M342 | Systemic sclerosis induced by drugs and chemicals |
| M357 | Hypermobility syndrome |
| M358 | Other specified systemic involvement of connective tissue |
| M359 | Systemic involvement of connective tissue, unspecified |
| M360 | Dermato(poly)myositis in neoplastic disease |
| M361 | Arthropathy in neoplastic disease |
| M362 | Haemophilic arthropathy |
| M363 | Arthropathy in other blood disorders |
| **BLINDNESS, VISUAL IMPAIRMENT** | |
| **Included ICD-10 codes and labels** | |
| H54 | Visual impairment including blindness (binocular or monocular) |
| Z442 | Fitting and adjustment of artificial eye |
| Z970 | Presence of artificial eye |
| **Excluded ICD-10 codes and labels** | |
| H543 | Mild or no visual impairment, binocular |
| **BLOOD AND BLOOD FORMING ORGAN DISEASES** | |
| **Included ICD-10 codes and labels** | |
| D66 | Hereditary factor VIII deficiency |
| D67 | Hereditary factor IX deficiency |
| D68 | Other coagulation defects |
| D69 | Purpura and other haemorrhagic conditions |
| D71 | Functional disorders of polymorphonuclear neutrophils |
| D720 | Genetic anomalies of leukocytes |
| D730 | Hyposplenism |
| D731 | Hypersplenism |
| D732 | Chronic congestive splenomegaly |
| D74 | Methaemoglobinaemia |
| D750 | Familial erythrocytosis |
| D761 | Haemophagocytic lymphohistiocytosis |
| D763 | Other histiocytosis syndromes |
| D77 | Other disorders of blood and blood-forming organs in diseases classified elsewhere |
| D80 | Immunodeficiency with predominantly antibody defects |
| D81 | Combined immunodeficiencies |
| D82 | Immunodeficiency associated with other major defects |
| D83 | Common variable immunodeficiency |
| D84 | Other immunodeficiencies |
| D86 | Sarcoidosis |
| D89 | Other disorders involving the immune mechanism, not elsewhere classified |
| **Excluded ICD-10 codes and labels** | |
| D683 | Haemorrhagic disorder due to circulating anticoagulants |
| D684 | Acquired coagulation factor deficiency |
| D695 | Secondary thrombocytopenia |
| D748 | Other methaemoglobinaemias |
| D807 | Transient hypogammaglobulinaemia of infancy |
| D891 | Cryoglobulinaemia |
| D893 | Immune reconstitution syndrome |
| **BRADYCARDIAS AND CONDUCTION DISEASES** | |
| **Included ICD-10 codes and labels** | |
| I441 | Atrioventricular block, second degree |
| I442 | Atrioventricular block, complete |
| I443 | Other and unspecified atrioventricular block |
| I453 | Trifascicular block |
| I455 | Other specified heart block |
| Z950 | Presence of cardiac pacemaker |
| **CARDIAC VALVE DISEASES** | |
| **Included ICD-10 codes and labels** | |
| I05 | Rheumatic mitral valve diseases |
| I06 | Rheumatic aortic valve diseases |
| I07 | Rheumatic tricuspid valve diseases |
| I08 | Multiple valve diseases |
| I091 | Rheumatic diseases of endocardium, valve unspecified |
| I098 | Other specified rheumatic heart diseases |
| I34 | Nonrheumatic mitral valve disorders |
| I35 | Nonrheumatic aortic valve disorders |
| I36 | Nonrheumatic tricuspid valve disorders |
| I37 | Pulmonary valve disorders |
| I38 | Endocarditis, valve unspecified |
| I390 | Mitral valve disorders in diseases classified elsewhere |
| I391 | Aortic valve disorders in diseases classified elsewhere |
| I392 | Tricuspid valve disorders in diseases classified elsewhere |
| I393 | Pulmonary valve disorders in diseases classified elsewhere |
| I394 | Multiple valve disorders in diseases classified elsewhere |
| Q22 | Congenital malformations of pulmonary and tricuspid valves |
| Q23 | Congenital malformations of aortic and mitral valves |
| Z952 | Presence of prosthetic heart valve |
| Z953 | Presence of xenogenic heart valve |
| Z954 | Presence of other heart-valve replacement |
| **CATARACT AND OTHER LENS DISEASES** | |
| **Included ICD-10 codes and labels** | |
| H25 | Senile cataract |
| H26 | Other cataract |
| H27 | Other disorders of lens |
| H28 | Cataract and other disorders of lens in diseases classified elsewhere |
| Q12 | Congenital lens malformations |
| Z961 | Presence of intraocular lens |
| **CEREBROVASCULAR DISEASE** | |
| **Included ICD-10 codes and labels** | |
| G45 | Transient cerebral ischaemic attacks and related syndromes |
| G46 | Vascular syndromes of brain in cerebrovascular diseases |
| I60 | Subarachnoid haemorrhage |
| I61 | Intracerebral haemorrhage |
| I62 | Other nontraumatic intracranial haemorrhage |
| I63 | Cerebral infarction |
| I64 | Stroke, not specified as haemorrhage or infarction |
| I67 | Other cerebrovascular diseases |
| I69 | Sequelae of cerebrovascular disease |
| **CHRONIC INFECTIOUS DISEASES** | |
| **Included ICD-10 codes and labels** | |
| A15 | Respiratory tuberculosis, bacteriologically and histologically confirmed |
| A16 | Respiratory tuberculosis, not confirmed bacteriologically or histologically |
| A17 | Tuberculosis of nervous system |
| A18 | Tuberculosis of other organs |
| A19 | Miliary tuberculosis |
| A30 | Leprosy [Hansen disease] |
| A31 | Infection due to other mycobacteria |
| A50 | Congenital syphilis |
| A52 | Late syphilis |
| A53 | Other and unspecified syphilis |
| A65 | Nonvenereal syphilis |
| A66 | Yaws |
| A67 | Pinta [carate] |
| A692 | Lyme disease |
| A81 | Atypical virus infections of central nervous system |
| B20 | Human immunodeficiency virus [HIV] disease resulting in infectious and parasitic diseases |
| B21 | Human immunodeficiency virus [HIV] disease resulting in malignant neoplasms |
| B22 | Human immunodeficiency virus [HIV] disease resulting in other specified diseases |
| B23 | Human immunodeficiency virus [HIV] disease resulting in other conditions |
| B24 | Unspecified human immunodeficiency virus [HIV] disease |
| B381 | Chronic pulmonary coccidioidomycosis |
| B391 | Chronic pulmonary histoplasmosis capsulati |
| B401 | Chronic pulmonary blastomycosis |
| B572 | Chagas disease (chronic) with heart involvement |
| B573 | Chagas disease (chronic) with digestive system involvement |
| B574 | Chagas disease (chronic) with nervous system involvement |
| B575 | Chagas disease (chronic) with other organ involvement |
| B65 | Schistosomiasis [bilharziasis] |
| B92 | Sequelae of leprosy |
| B94 | Sequelae of other and unspecified infectious and parasitic diseases |
| J65 | Pneumoconiosis associated with tuberculosis |
| M863 | Chronic multifocal osteomyelitis |
| M864 | Chronic osteomyelitis with draining sinus |
| M865 | Other chronic haematogenous osteomyelitis |
| M866 | Other chronic osteomyelitis |
| **CHRONIC KIDNEY DISEASES** | |
| **Included ICD-10 codes and labels** | |
| I120 | Hypertensive renal disease with renal failure |
| I130 | Hypertensive heart and renal disease with (congestive) heart failure |
| I131 | Hypertensive heart and renal disease with renal failure |
| I132 | Hypertensive heart and renal disease with both (congestive) heart failure and renal failure |
| I139 | Hypertensive heart and renal disease, unspecified |
| N01 | Rapidly progressive nephritic syndrome |
| N03 | Chronic nephritic syndrome |
| N04 | Nephrotic syndrome |
| N05 | Unspecified nephritic syndrome |
| N07 | Hereditary nephropathy, not elsewhere classified |
| N08 | Glomerular disorders in diseases classified elsewhere |
| N11 | Chronic tubulo-interstitial nephritis |
| N183 | Chronic kidney disease, stage 3 |
| N184 | Chronic kidney disease, stage 4 |
| N185 | Chronic kidney disease, stage 5 |
| N189 | Chronic kidney disease, unspecified |
| Q60 | Renal agenesis and other reduction defects of kidney |
| Q611 | Polycystic kidney, autosomal recessive |
| Q612 | Polycystic kidney, autosomal dominant |
| Q613 | Polycystic kidney, unspecified |
| Q614 | Renal dysplasia |
| Q615 | Medullary cystic kidney |
| Q618 | Other cystic kidney diseases |
| Q619 | Cystic kidney disease, unspecified |
| Z905 | Acquired absence of kidney |
| Z940 | Kidney transplant status |
| **CHRONIC LIVER DISEASES** | |
| **Included ICD-10 codes and labels** | |
| B18 | Chronic viral hepatitis |
| K70 | Alcoholic liver disease |
| K713 | Toxic liver disease with chronic persistent hepatitis |
| K714 | Toxic liver disease with chronic lobular hepatitis |
| K715 | Toxic liver disease with chronic active hepatitis |
| K717 | Toxic liver disease with fibrosis and cirrhosis of liver |
| K721 | Chronic hepatic failure |
| K73 | Chronic hepatitis, not elsewhere classified |
| K74 | Fibrosis and cirrhosis of liver |
| K753 | Granulomatous hepatitis, not elsewhere classified |
| K754 | Autoimmune hepatitis |
| K758 | Other specified inflammatory liver diseases |
| K761 | Chronic passive congestion of liver |
| K766 | Portal hypertension |
| K767 | Hepatorenal syndrome |
| K778 | Liver disorders in other diseases classified elsewhere |
| Q446 | Cystic disease of liver |
| Z944 | Liver transplant status |
| **Excluded ICD-10 codes and labels** | |
| K700 | Alcoholic fatty liver |
| K701 | Alcoholic hepatitis |
| **CHRONIC PANCREAS, BILIARY TRACT AND GALLBLADDER DISEASES** | |
| **Included ICD-10 codes and labels** | |
| K800 | Calculus of gallbladder with acute cholecystitis |
| K801 | Calculus of gallbladder with other cholecystitis |
| K802 | Calculus of gallbladder without cholecystitis |
| K808 | Other cholelithiasis |
| K811 | Chronic cholecystitis |
| K86 | Other diseases of pancreas |
| Q440 | Agenesis, aplasia and hypoplasia of gallbladder |
| Q441 | Other congenital malformations of gallbladder |
| Q442 | Atresia of bile ducts |
| Q443 | Congenital stenosis and stricture of bile ducts |
| Q444 | Choledochal cyst |
| Q445 | Other congenital malformations of bile ducts |
| Q450 | Agenesis, aplasia and hypoplasia of pancreas |
| **Excluded ICD-10 codes and labels** | |
| K862 | Cyst of pancreas |
| K863 | Pseudocyst of pancreas |
| K869 | Disease of pancreas, unspecified |
| **CHRONIC ULCER OF THE SKIN** | |
| **Included ICD-10 codes and labels** | |
| I830 | Varicose veins of lower extremities with ulcer |
| I832 | Varicose veins of lower extremities with both ulcer and inflammation |
| L89 | Decubitus ulcer and pressure area |
| L97 | Ulcer of lower limb, not elsewhere classified |
| L984 | Chronic ulcer of skin, not elsewhere classified |
| **COLITIS AND RELATED DISEASES** | |
| **Included ICD-10 codes and labels** | |
| K520 | Gastroenteritis and colitis due to radiation |
| K528 | Other specified noninfective gastroenteritis and colitis |
| K551 | Chronic vascular disorders of intestine |
| K552 | Angiodysplasia of colon |
| K572 | Diverticular disease of large intestine with perforation and abscess |
| K573 | Diverticular disease of large intestine without perforation or abscess |
| K574 | Diverticular disease of both small and large intestine with perforation and abscess |
| K575 | Diverticular disease of both small and large intestine without perforation or abscess |
| K578 | Diverticular disease of intestine, part unspecified, with perforation and abscess |
| K579 | Diverticular disease of intestine, part unspecified, without perforation or abscess |
| K58 | Irritable bowel syndrome |
| K590 | Constipation |
| K592 | Neurogenic bowel, not elsewhere classified |
| K62 | Other diseases of anus and rectum |
| K634 | Enteroptosis |
| K64 | Haemorrhoids and perianal venous thrombosis |
| **Excluded ICD-10 codes and labels** | |
| K620 | Anal polyp |
| K621 | Rectal polyp |
| K625 | Haemorrhage of anus and rectum |
| K626 | Ulcer of anus and rectum |
| K645 | Perianal venous thrombosis |
| **COPD, EMPHYSEMA, CHRONIC BRONCHITIS** | |
| **Included ICD-10 codes and labels** | |
| J41 | Simple and mucopurulent chronic bronchitis |
| J42 | Unspecified chronic bronchitis |
| J43 | Emphysema |
| J44 | Other chronic obstructive pulmonary disease |
| J47 | Bronchiectasis |
| **DEAFNESS, HEARING IMPAIRMENT** | |
| **Included ICD-10 codes and labels** | |
| H80 | Otosclerosis |
| H90 | Conductive and sensorineural hearing loss |
| H911 | Presbycusis |
| H913 | Deaf mutism, not elsewhere classified |
| H919 | Hearing loss, unspecified |
| Q16 | Congenital malformations of ear causing impairment of hearing |
| Z453 | Adjustment and management of implanted hearing device |
| Z461 | Fitting and adjustment of hearing aid |
| Z962 | Presence of otological and audiological implants |
| Z974 | Presence of external hearing-aid |
| **DEMENTIA** | |
| **Included ICD-10 codes and labels** | |
| F00 | Dementia in Alzheimer disease |
| F01 | Vascular dementia |
| F02 | Dementia in other diseases classified elsewhere |
| F03 | Unspecified dementia |
| F051 | Delirium superimposed on dementia |
| G30 | Alzheimer disease |
| G31 | Other degenerative diseases of nervous system, not elsewhere classified |
| **DEPRESSION AND MOOD DISEASES** | |
| **Included ICD-10 codes and labels** | |
| F30 | Manic episode |
| F31 | Bipolar affective disorder |
| F32 | Depressive episode |
| F33 | Recurrent depressive disorder |
| F34 | Persistent mood [affective] disorders |
| F38 | Other mood [affective] disorders |
| F39 | Unspecified mood [affective] disorder |
| F412 | Mixed anxiety and depressive disorder |
| **DIABETES** | |
| **Included ICD-10 codes and labels** | |
| E10 | Insulin-dependent diabetes mellitus |
| E11 | Non-insulin-dependent diabetes mellitus |
| E13 | Other specified diabetes mellitus |
| E14 | Unspecified diabetes mellitus |
| E891 | Postprocedural hypoinsulinaemia |
| **DORSOPATHIES** | |
| **Included ICD-10 codes and labels** | |
| M40 | Kyphosis and lordosis |
| M41 | Scoliosis |
| M42 | Spinal osteochondrosis |
| M43 | Other deforming dorsopathies |
| M47 | Spondylosis |
| M48 | Other spondylopathies |
| M49 | Spondylopathies in diseases classified elsewhere |
| M50 | Cervical disc disorders |
| M51 | Other intervertebral disc disorders |
| M53 | Other dorsopathies, not elsewhere classified |
| Q675 | Congenital deformity of spine |
| Q761 | Klippel-Feil syndrome |
| Q764 | Other congenital malformations of spine, not associated with scoliosis |
| **DYSLIPIDEMIA** | |
| **Included ICD-10 codes and labels** | |
| E78 | Disorders of lipoprotein metabolism and other lipidaemias |
| **EAR, NOSE, THROAT DISEASES** | |
| **Included ICD-10 codes and labels** | |
| H604 | Cholesteatoma of external ear |
| H661 | Chronic tubotympanic suppurative otitis media |
| H662 | Chronic atticoantral suppurative otitis media |
| H663 | Other chronic suppurative otitis media |
| H701 | Chronic mastoiditis |
| H71 | Cholesteatoma of middle ear |
| H731 | Chronic myringitis |
| H741 | Adhesive middle ear disease |
| H810 | MÚniÞre disease |
| H831 | Labyrinthine fistula |
| H832 | Labyrinthine dysfunction |
| H95 | Postprocedural disorders of ear and mastoid process, not elsewhere classified |
| J300 | Vasomotor rhinitis |
| J31 | Chronic rhinitis, nasopharyngitis and pharyngitis |
| J32 | Chronic sinusitis |
| J33 | Nasal polyp |
| J341 | Cyst and mucocele of nose and nasal sinus |
| J342 | Deviated nasal septum |
| J343 | Hypertrophy of nasal turbinates |
| J35 | Chronic diseases of tonsils and adenoids |
| J37 | Chronic laryngitis and laryngotracheitis |
| J380 | Paralysis of vocal cords and larynx |
| J386 | Stenosis of larynx |
| K051 | Chronic gingivitis |
| K053 | Chronic periodontitis |
| K07 | Dentofacial anomalies [including malocclusion] |
| K110 | Atrophy of salivary gland |
| K117 | Disturbances of salivary secretion |
| Q30 | Congenital malformations of nose |
| Q31 | Congenital malformations of larynx |
| Q32 | Congenital malformations of trachea and bronchus |
| Q35 | Cleft palate |
| Q36 | Cleft lip |
| Q37 | Cleft palate with cleft lip |
| Q38 | Other congenital malformations of tongue, mouth and pharynx |
| **EPILEPSY** | |
| **Included ICD-10 codes and labels** | |
| G40 | Epilepsy |
| **Excluded ICD-10 codes and labels** | |
| G405 | Special epileptic syndromes |
| **ESOPHAGUS, STOMACH AND DUODENUM DISEASES** | |
| **Included ICD-10 codes and labels** | |
| I85 | Oesophageal varices |
| I864 | Gastric varices |
| I982 | Oesophageal varices without bleeding in diseases classified elsewhere |
| I983 | Oesophageal varices with bleeding in diseases classified elsewhere |
| K21 | Gastro-oesophageal reflux disease |
| K220 | Achalasia of cardia |
| K222 | Oesophageal obstruction |
| K224 | Dyskinesia of oesophagus |
| K225 | Diverticulum of oesophagus, acquired |
| K227 | Barrett oesophagus |
| K230 | Tuberculous oesophagitis |
| K231 | Megaoesophagus in Chagas disease |
| K254 | Gastric ulcer: Chronic or unspecified with haemorrhage |
| K255 | Gastric ulcer: Chronic or unspecified with perforation |
| K256 | Gastric ulcer: Chronic or unspecified with both haemorrhage and perforation |
| K257 | Gastric ulcer: Chronic without haemorrhage or perforation |
| K264 | Duodenal ulcer: Chronic or unspecified with haemorrhage |
| K265 | Duodenal ulcer: Chronic or unspecified with perforation |
| K266 | Duodenal ulcer: Chronic or unspecified with both haemorrhage and perforation |
| K267 | Duodenal ulcer: Chronic without haemorrhage or perforation |
| K274 | Peptic ulcer, site unspecified: Chronic or unspecified with haemorrhage |
| K275 | Peptic ulcer, site unspecified: Chronic or unspecified with perforation |
| K276 | Peptic ulcer, site unspecified: Chronic or unspecified with both haemorrhage and perforation |
| K277 | Peptic ulcer, site unspecified: Chronic without haemorrhage or perforation |
| K284 | Gastrojejunal ulcer: Chronic or unspecified with haemorrhage |
| K285 | Gastrojejunal ulcer: Chronic or unspecified with perforation |
| K286 | Gastrojejunal ulcer: Chronic or unspecified with both haemorrhage and perforation |
| K287 | Gastrojejunal ulcer: Chronic without haemorrhage or perforation |
| K293 | Chronic superficial gastritis |
| K294 | Chronic atrophic gastritis |
| K295 | Chronic gastritis, unspecified |
| K296 | Other gastritis |
| K297 | Gastritis, unspecified |
| K298 | Duodenitis |
| K299 | Gastroduodenitis, unspecified |
| K311 | Adult hypertrophic pyloric stenosis |
| K312 | Hourglass stricture and stenosis of stomach |
| K313 | Pylorospasm, not elsewhere classified |
| K314 | Gastric diverticulum |
| K315 | Obstruction of duodenum |
| Q39 | Congenital malformations of oesophagus |
| Q40 | Other congenital malformations of upper alimentary tract |
| Z903 | Acquired absence of part of stomach |
| **GLAUCOMA** | |
| **Included ICD-10 codes and labels** | |
| H401 | Primary open-angle glaucoma |
| H402 | Primary angle-closure glaucoma |
| H403 | Glaucoma secondary to eye trauma |
| H404 | Glaucoma secondary to eye inflammation |
| H405 | Glaucoma secondary to other eye disorders |
| H406 | Glaucoma secondary to drugs |
| H408 | Other glaucoma |
| H409 | Glaucoma, unspecified |
| **HEART FAILURE** | |
| **Included ICD-10 codes and labels** | |
| I110 | Hypertensive heart disease with (congestive) heart failure |
| I130 | Hypertensive heart and renal disease with (congestive) heart failure |
| I132 | Hypertensive heart and renal disease with both (congestive) heart failure and renal failure |
| I27 | Other pulmonary heart diseases |
| I280 | Arteriovenous fistula of pulmonary vessels |
| I42 | Cardiomyopathy |
| I43 | Cardiomyopathy in diseases classified elsewhere |
| I50 | Heart failure |
| I515 | Myocardial degeneration |
| I517 | Cardiomegaly |
| I528 | Other heart disorders in other diseases classified elsewhere |
| Z941 | Heart transplant status |
| Z943 | Heart and lungs transplant status |
| **HEMATOLOGICAL NEOPLASMS** | |
| **Included ICD-10 codes and labels** | |
| C81 | Hodgkin lymphoma |
| C82 | Follicular lymphoma |
| C83 | Non-follicular lymphoma |
| C84 | Mature T/NK-cell lymphomas |
| C85 | Other and unspecified types of non-Hodgkin lymphoma |
| C86 | Other specified types of T/NK-cell lymphoma |
| C88 | Malignant immunoproliferative diseases |
| C90 | Multiple myeloma and malignant plasma cell neoplasms |
| C91 | Lymphoid leukaemia |
| C92 | Myeloid leukaemia |
| C93 | Monocytic leukaemia |
| C94 | Other leukaemias of specified cell type |
| C95 | Leukaemia of unspecified cell type |
| C96 | Other and unspecified malignant neoplasms of lymphoid, haematopoietic and related tissue |
| **HYPERTENSION** | |
| **Included ICD-10 codes and labels** | |
| I10 | Essential (primary) hypertension |
| I11 | Hypertensive heart disease |
| I12 | Hypertensive renal disease |
| I13 | Hypertensive heart and renal disease |
| I15 | Secondary hypertension |
| **INFLAMMATORY ARTHROPATHIES** | |
| **Included ICD-10 codes and labels** | |
| M023 | Reiter disease |
| M05 | Seropositive rheumatoid arthritis |
| M06 | Other rheumatoid arthritis |
| M07 | Psoriatic and enteropathic arthropathies |
| M08 | Juvenile arthritis |
| M09 | Juvenile arthritis in diseases classified elsewhere |
| M10 | Gout |
| M11 | Other crystal arthropathies |
| M12 | Other specific arthropathies |
| M13 | Other arthritis |
| M14 | Arthropathies in other diseases classified elsewhere |
| M45 | Ankylosing spondylitis |
| M460 | Spinal enthesopathy |
| M461 | Sacroiliitis, not elsewhere classified |
| M468 | Other specified inflammatory spondylopathies |
| M469 | Inflammatory spondylopathy, unspecified |
| **INFLAMMATORY BOWEL DISEASES** | |
| **Included ICD-10 codes and labels** | |
| K50 | Crohn disease [regional enteritis] |
| K51 | Ulcerative colitis |
| **ISCHEMIC HEART DISEASE** | |
| **Included ICD-10 codes and labels** | |
| I20 | Angina pectoris |
| I21 | Acute myocardial infarction |
| I22 | Subsequent myocardial infarction |
| I24 | Other acute ischaemic heart diseases |
| I25 | Chronic ischaemic heart disease |
| Z951 | Presence of aortocoronary bypass graft |
| Z955 | Presence of coronary angioplasty implant and graft |
| **MIGRAINE AND FACIAL PAIN SYNDROMES** | |
| **Included ICD-10 codes and labels** | |
| G43 | Migraine |
| G440 | Cluster headache syndrome |
| G441 | Vascular headache, not elsewhere classified |
| G442 | Tension-type headache |
| G443 | Chronic post-traumatic headache |
| G448 | Other specified headache syndromes |
| G50 | Disorders of trigeminal nerve |
| **MULTIPLE SCLEROSIS** | |
| **Included ICD-10 codes and labels** | |
| G35 | Multiple sclerosis |
| **NEUROTIC, STRESS-RELATED AND SOMATOFORM DISEASES** | |
| **Included ICD-10 codes and labels** | |
| F40 | Phobic anxiety disorders |
| F41 | Other anxiety disorders |
| F42 | Obsessive-compulsive disorder |
| F43 | Reaction to severe stress, and adjustment disorders |
| F44 | Dissociative [conversion] disorders |
| F45 | Somatoform disorders |
| F48 | Other neurotic disorders |
| **Excluded ICD-10 codes and labels** | |
| F430 | Acute stress reaction |
| F432 | Adjustment disorders |
| **OBESITY** | |
| **Included ICD-10 codes and labels** | |
| E66 | Obesity |
| **OSTEOARTHRITIS AND OTHER DEGENERATIVE JOINT DISEASES** | |
| **Included ICD-10 codes and labels** | |
| M15 | Polyarthrosis |
| M16 | Coxarthrosis [arthrosis of hip] |
| M17 | Gonarthrosis [arthrosis of knee] |
| M18 | Arthrosis of first carpometacarpal joint |
| M19 | Other arthrosis |
| M362 | Haemophilic arthropathy |
| M363 | Arthropathy in other blood disorders |
| **OSTEOPOROSIS** | |
| **Included ICD-10 codes and labels** | |
| M80 | Osteoporosis with pathological fracture |
| M81 | Osteoporosis without pathological fracture |
| M82 | Osteoporosis in diseases classified elsewhere |
| **OTHER CARDIOVASCULAR DISEASES** | |
| **Included ICD-10 codes and labels** | |
| I09 | Other rheumatic heart diseases |
| I281 | Aneurysm of pulmonary artery |
| I310 | Chronic adhesive pericarditis |
| I311 | Chronic constrictive pericarditis |
| I456 | Pre-excitation syndrome |
| I495 | Sick sinus syndrome |
| I498 | Other specified cardiac arrhythmias |
| I70 | Atherosclerosis |
| I71 | Aortic aneurysm and dissection |
| I72 | Other aneurysm and dissection |
| I790 | Aneurysm of aorta in diseases classified elsewhere |
| I791 | Aortitis in diseases classified elsewhere |
| I950 | Idiopathic hypotension |
| I951 | Orthostatic hypotension |
| I958 | Other hypotension |
| Q20 | Congenital malformations of cardiac chambers and connections |
| Q21 | Congenital malformations of cardiac septa |
| Q24 | Other congenital malformations of heart |
| Q25 | Congenital malformations of great arteries |
| Q26 | Congenital malformations of great veins |
| Q27 | Other congenital malformations of peripheral vascular system |
| Q28 | Other congenital malformations of circulatory system |
| Z958 | Presence of other cardiac and vascular implants and grafts |
| Z959 | Presence of cardiac and vascular implant and graft, unspecified |
| **Excluded ICD-10 codes and labels** | |
| I091 | Rheumatic diseases of endocardium, valve unspecified |
| I098 | Other specified rheumatic heart diseases |
| I702 | Atherosclerosis of arteries of extremities |
| **OTHER DIGESTIVE DISEASES** | |
| **Included ICD-10 codes and labels** | |
| K660 | Peritoneal adhesions |
| K900 | Coeliac disease |
| K901 | Tropical sprue |
| K902 | Blind loop syndrome, not elsewhere classified |
| K911 | Postgastric surgery syndromes |
| K93 | Disorders of other digestive organs in diseases classified elsewhere |
| Q41 | Congenital absence, atresia and stenosis of small intestine |
| Q42 | Congenital absence, atresia and stenosis of large intestine |
| Q43 | Other congenital malformations of intestine |
| R15 | Faecal incontinence |
| Z904 | Acquired absence of other parts of digestive tract |
| Z980 | Intestinal bypass and anastomosis status |
| **OTHER EYE DISEASES** | |
| **Included ICD-10 codes and labels** | |
| H022 | Lagophthalmos |
| H023 | Blepharochalasis |
| H024 | Ptosis of eyelid |
| H025 | Other disorders affecting eyelid function |
| H04 | Disorders of lacrimal system |
| H05 | Disorders of orbit |
| H104 | Chronic conjunctivitis |
| H17 | Corneal scars and opacities |
| H184 | Corneal degeneration |
| H185 | Hereditary corneal dystrophies |
| H186 | Keratoconus |
| H187 | Other corneal deformities |
| H188 | Other specified disorders of cornea |
| H189 | Disorder of cornea, unspecified |
| H193 | Keratitis and keratoconjunctivitis in other diseases classified elsewhere |
| H198 | Other disorders of sclera and cornea in diseases classified elsewhere |
| H201 | Chronic iridocyclitis |
| H21 | Other disorders of iris and ciliary body |
| H310 | Chorioretinal scars |
| H311 | Choroidal degeneration |
| H312 | Hereditary choroidal dystrophy |
| H318 | Other specified disorders of choroid |
| H319 | Disorder of choroid, unspecified |
| H33 | Retinal detachments and breaks |
| H352 | Other proliferative retinopathy |
| H353 | Degeneration of macula and posterior pole |
| H354 | Peripheral retinal degeneration |
| H355 | Hereditary retinal dystrophy |
| H357 | Separation of retinal layers |
| H358 | Other specified retinal disorders |
| H359 | Retinal disorder, unspecified |
| H36 | Retinal disorders in diseases classified elsewhere |
| H47 | Other disorders of optic [2nd] nerve and visual pathways |
| H48 | Disorders of optic [2nd] nerve and visual pathways in diseases classified elsewhere |
| H49 | Paralytic strabismus |
| H51 | Other disorders of binocular movement |
| Q10 | Congenital malformations of eyelid, lacrimal apparatus and orbit |
| Q11 | Anophthalmos, microphthalmos and macrophthalmos |
| Q13 | Congenital malformations of anterior segment of eye |
| Q14 | Congenital malformations of posterior segment of eye |
| Q15 | Other congenital malformations of eye |
| Z947 | Corneal transplant status |
| **Excluded ICD-10 codes and labels** | |
| H043 | Acute and unspecified inflammation of lacrimal passages |
| H050 | Acute inflammation of orbit |
| H470 | Disorders of optic nerve, not elsewhere classified |
| H471 | Papilloedema, unspecified |
| H481 | Retrobulbar neuritis in diseases classified elsewhere |
| **OTHER GENITOURINARY DISEASES** | |
| **Included ICD-10 codes and labels** | |
| B901 | Sequelae of genitourinary tuberculosis |
| N200 | Calculus of kidney |
| N202 | Calculus of kidney with calculus of ureter |
| N209 | Urinary calculus, unspecified |
| N210 | Calculus in bladder |
| N218 | Other lower urinary tract calculus |
| N219 | Calculus of lower urinary tract, unspecified |
| N22 | Calculus of urinary tract in diseases classified elsewhere |
| N301 | Interstitial cystitis (chronic) |
| N302 | Other chronic cystitis |
| N303 | Trigonitis |
| N304 | Irradiation cystitis |
| N31 | Neuromuscular dysfunction of bladder, not elsewhere classified |
| N320 | Bladder-neck obstruction |
| N323 | Diverticulum of bladder |
| N328 | Other specified disorders of bladder |
| N329 | Bladder disorder, unspecified |
| N33 | Bladder disorders in diseases classified elsewhere |
| N35 | Urethral stricture |
| N393 | Stress incontinence |
| N394 | Other specified urinary incontinence |
| N480 | Leukoplakia of penis |
| N484 | Impotence of organic origin |
| N489 | Disorder of penis, unspecified |
| N701 | Chronic salpingitis and oophoritis |
| N711 | Chronic inflammatory disease of uterus |
| N731 | Chronic parametritis and pelvic cellulitis |
| N734 | Female chronic pelvic peritonitis |
| N736 | Female pelvic peritoneal adhesions |
| N761 | Subacute and chronic vaginitis |
| N763 | Subacute and chronic vulvitis |
| N81 | Female genital prolapse |
| N88 | Other noninflammatory disorders of cervix uteri |
| N895 | Stricture and atresia of vagina |
| N905 | Atrophy of vulva |
| N952 | Postmenopausal atrophic vaginitis |
| Q54 | Hypospadias |
| Q620 | Congenital hydronephrosis |
| Q621 | Atresia and stenosis of ureter |
| Q622 | Congenital megaloureter |
| Q623 | Other obstructive defects of renal pelvis and ureter |
| Q624 | Agenesis of ureter |
| Q627 | Congenital vesico-uretero-renal reflux |
| Q628 | Other congenital malformations of ureter |
| Q638 | Other specified congenital malformations of kidney |
| Q639 | Congenital malformation of kidney, unspecified |
| Q640 | Epispadias |
| Q641 | Exstrophy of urinary bladder |
| Q643 | Other atresia and stenosis of urethra and bladder neck |
| Q644 | Malformation of urachus |
| Q645 | Congenital absence of bladder and urethra |
| Q646 | Congenital diverticulum of bladder |
| Q647 | Other congenital malformations of bladder and urethra |
| Q648 | Other specified congenital malformations of urinary system |
| Q649 | Congenital malformation of urinary system, unspecified |
| Z906 | Acquired absence of other organs of urinary tract |
| Z907 | Acquired absence of genital organ(s) |
| Z960 | Presence of urogenital implants |
| **OTHER METABOLIC DISEASES** | |
| **Included ICD-10 codes and labels** | |
| E20 | Hypoparathyroidism |
| E21 | Hyperparathyroidism and other disorders of parathyroid gland |
| E22 | Hyperfunction of pituitary gland |
| E23 | Hypofunction and other disorders of pituitary gland |
| E24 | Cushing syndrome |
| E25 | Adrenogenital disorders |
| E26 | Hyperaldosteronism |
| E27 | Other disorders of adrenal gland |
| E28 | Ovarian dysfunction |
| E29 | Testicular dysfunction |
| E31 | Polyglandular dysfunction |
| E34 | Other endocrine disorders |
| E35 | Disorders of endocrine glands in diseases classified elsewhere |
| E40 | Kwashiorkor |
| E41 | Nutritional marasmus |
| E42 | Marasmic kwashiorkor |
| E43 | Unspecified severe protein-energy malnutrition |
| E44 | Protein-energy malnutrition of moderate and mild degree |
| E45 | Retarded development following protein-energy malnutrition |
| E46 | Unspecified protein-energy malnutrition |
| E64 | Sequelae of malnutrition and other nutritional deficiencies |
| E70 | Disorders of aromatic amino-acid metabolism |
| E71 | Disorders of branched-chain amino-acid metabolism and fatty-acid metabolism |
| E72 | Other disorders of amino-acid metabolism |
| E74 | Other disorders of carbohydrate metabolism |
| E75 | Disorders of sphingolipid metabolism and other lipid storage disorders |
| E76 | Disorders of glycosaminoglycan metabolism |
| E77 | Disorders of glycoprotein metabolism |
| E79 | Disorders of purine and pyrimidine metabolism |
| E80 | Disorders of porphyrin and bilirubin metabolism |
| E83 | Disorders of mineral metabolism |
| E84 | Cystic fibrosis |
| E85 | Amyloidosis |
| E88 | Other metabolic disorders |
| E89 | Postprocedural endocrine and metabolic disorders, not elsewhere classified |
| K903 | Pancreatic steatorrhoea |
| K904 | Malabsorption due to intolerance, not elsewhere classified |
| K908 | Other intestinal malabsorption |
| K909 | Intestinal malabsorption, unspecified |
| K912 | Postsurgical malabsorption, not elsewhere classified |
| M83 | Adult osteomalacia |
| M88 | Paget disease of bone [osteitis deformans] |
| N25 | Disorders resulting from impaired renal tubular function |
| **Excluded ICD-10 codes and labels** | |
| E231 | Drug-induced hypopituitarism |
| E242 | Drug-induced Cushing syndrome |
| E244 | Alcohol-induced pseudo-Cushing syndrome |
| E273 | Drug-induced adrenocortical insufficiency |
| E343 | Short stature, not elsewhere classified |
| E344 | Constitutional tall stature |
| E350 | Disorders of thyroid gland in diseases classified elsewhere |
| E441 | Mild protein-energy malnutrition |
| E790 | Hyperuricaemia without signs of inflammatory arthritis and tophaceous disease |
| E804 | Gilbert syndrome |
| E883 | Tumour lysis syndrome |
| E890 | Postprocedural hypothyroidism |
| E892 | Postprocedural hypoparathyroidism |
| **OTHER MUSCULOSKELETAL AND JOINT DISEASES** | |
| **Included ICD-10 codes and labels** | |
| B902 | Sequelae of tuberculosis of bones and joints |
| M212 | Flexion deformity |
| M213 | Wrist or foot drop (acquired) |
| M214 | Flat foot [pes planus] (acquired) |
| M215 | Acquired clawhand, clubhand, clawfoot and clubfoot |
| M216 | Other acquired deformities of ankle and foot |
| M217 | Unequal limb length (acquired) |
| M218 | Other specified acquired deformities of limbs |
| M219 | Acquired deformity of limb, unspecified |
| M22 | Disorders of patella |
| M23 | Internal derangement of knee |
| M24 | Other specific joint derangements |
| M252 | Flail joint |
| M253 | Other instability of joint |
| M357 | Hypermobility syndrome |
| M61 | Calcification and ossification of muscle |
| M652 | Calcific tendinitis |
| M653 | Trigger finger |
| M654 | Radial styloid tenosynovitis [de Quervain] |
| M700 | Chronic crepitant synovitis of hand and wrist |
| M720 | Palmar fascial fibromatosis [Dupuytren] |
| M722 | Plantar fascial fibromatosis |
| M724 | Pseudosarcomatous fibromatosis |
| M750 | Adhesive capsulitis of shoulder |
| M751 | Rotator cuff syndrome |
| M753 | Calcific tendinitis of shoulder |
| M754 | Impingement syndrome of shoulder |
| M797 | Fibromyalgia |
| M841 | Nonunion of fracture [pseudarthrosis] |
| M89 | Other disorders of bone |
| M91 | Juvenile osteochondrosis of hip and pelvis |
| M93 | Other osteochondropathies |
| M94 | Other disorders of cartilage |
| M96 | Postprocedural musculoskeletal disorders, not elsewhere classified |
| M99 | Biomechanical lesions, not elsewhere classified |
| Q65 | Congenital deformities of hip |
| Q66 | Congenital deformities of feet |
| Q68 | Other congenital musculoskeletal deformities |
| Q71 | Reduction defects of upper limb |
| Q72 | Reduction defects of lower limb |
| Q73 | Reduction defects of unspecified limb |
| Q74 | Other congenital malformations of limb(s) |
| Q77 | Osteochondrodysplasia with defects of growth of tubular bones and spine |
| Q78 | Other osteochondrodysplasias |
| Q796 | Ehlers-Danlos syndrome |
| Q798 | Other congenital malformations of musculoskeletal system |
| Q87 | Other specified congenital malformation syndromes affecting multiple systems |
| S382 | Traumatic amputation of external genital organs |
| S48 | Traumatic amputation of shoulder and upper arm |
| S58 | Traumatic amputation of forearm |
| S68 | Traumatic amputation of wrist and hand |
| S78 | Traumatic amputation of hip and thigh |
| S88 | Traumatic amputation of lower leg |
| S98 | Traumatic amputation of ankle and foot |
| T05 | Traumatic amputations involving multiple body regions |
| T096 | Traumatic amputation of trunk, level unspecified |
| T116 | Traumatic amputation of upper limb, level unspecified |
| T136 | Traumatic amputation of lower limb, level unspecified |
| T147 | Crushing injury and traumatic amputation of unspecified body region |
| T90 | Sequelae of injuries of head |
| T91 | Sequelae of injuries of neck and trunk |
| T92 | Sequelae of injuries of upper limb |
| T93 | Sequelae of injuries of lower limb |
| T94 | Sequelae of injuries involving multiple and unspecified body regions |
| T95 | Sequelae of burns, corrosions and frostbite |
| T96 | Sequelae of poisoning by drugs, medicaments and biological substances |
| T97 | Sequelae of toxic effects of substances chiefly nonmedicinal as to source |
| T98 | Sequelae of other and unspecified effects of external causes |
| Z440 | Fitting and adjustment of artificial arm (complete)(partial) |
| Z441 | Fitting and adjustment of artificial leg (complete)(partial) |
| Z891 | Acquired absence of hand and wrist |
| Z892 | Acquired absence of upper limb above wrist |
| Z893 | Acquired absence of both upper limbs [any level] |
| Z894 | Acquired absence of foot and ankle |
| Z895 | Acquired absence of leg at or below knee |
| Z896 | Acquired absence of leg above knee |
| Z897 | Acquired absence of both lower limbs [any level, except toes alone] |
| Z898 | Acquired absence of upper and lower limbs [any level] |
| Z899 | Acquired absence of limb, unspecified |
| Z946 | Bone transplant status |
| Z966 | Presence of orthopaedic joint implants |
| Z971 | Presence of artificial limb (complete)(partial) |
| **OTHER NEUROLOGICAL DISEASES** | |
| **Included ICD-10 codes and labels** | |
| B900 | Sequelae of central nervous system tuberculosis |
| D482 | Neoplasm of uncertain or unknown behaviour: Peripheral nerves and autonomic nervous system |
| G041 | Tropical spastic paraplegia |
| G09 | Sequelae of inflammatory diseases of central nervous system |
| G10 | Huntington disease |
| G11 | Hereditary ataxia |
| G12 | Spinal muscular atrophy and related syndromes |
| G13 | Systemic atrophies primarily affecting central nervous system in diseases classified elsewhere |
| G24 | Dystonia |
| G25 | Other extrapyramidal and movement disorders |
| G26 | Extrapyramidal and movement disorders in diseases classified elsewhere |
| G32 | Other degenerative disorders of nervous system in diseases classified elsewhere |
| G37 | Other demyelinating diseases of central nervous system |
| G51 | Facial nerve disorders |
| G52 | Disorders of other cranial nerves |
| G53 | Cranial nerve disorders in diseases classified elsewhere |
| G70 | Myasthenia gravis and other myoneural disorders |
| G71 | Primary disorders of muscles |
| G723 | Periodic paralysis |
| G724 | Inflammatory myopathy, not elsewhere classified |
| G728 | Other specified myopathies |
| G729 | Myopathy, unspecified |
| G73 | Disorders of myoneural junction and muscle in diseases classified elsewhere |
| G80 | Cerebral palsy |
| G81 | Hemiplegia |
| G82 | Paraplegia and tetraplegia |
| G83 | Other paralytic syndromes |
| G90 | Disorders of autonomic nervous system |
| G91 | Hydrocephalus |
| G938 | Other specified disorders of brain |
| G939 | Disorder of brain, unspecified |
| G95 | Other diseases of spinal cord |
| G99 | Other disorders of nervous system in diseases classified elsewhere |
| M471 | Other spondylosis with myelopathy |
| Q00 | Anencephaly and similar malformations |
| Q01 | Encephalocele |
| Q02 | Microcephaly |
| Q03 | Congenital hydrocephalus |
| Q04 | Other congenital malformations of brain |
| Q05 | Spina bifida |
| Q06 | Other congenital malformations of spinal cord |
| Q07 | Other congenital malformations of nervous system |
| Q760 | Spina bifida occulta |
| **Excluded ICD-10 codes and labels** | |
| G130 | Paraneoplastic neuromyopathy and neuropathy |
| G131 | Other systemic atrophy primarily affecting central nervous system in neoplastic disease |
| G251 | Drug-induced tremor |
| G254 | Drug-induced chorea |
| G256 | Drug-induced tics and other tics of organic origin |
| G510 | Bell palsy |
| G732 | Other myasthenic syndromes in neoplastic disease |
| G733 | Myasthenic syndromes in other diseases classified elsewhere |
| G734 | Myopathy in infectious and parasitic diseases classified elsewhere |
| G838 | Other specified paralytic syndromes |
| **OTHER PSYCHIATRIC AND BEHAVIORAL DISEASES** | |
| **Included ICD-10 codes and labels** | |
| F04 | Organic amnesic syndrome, not induced by alcohol and other psychoactive substances |
| F06 | Other mental disorders due to brain damage and dysfunction and to physical disease |
| F07 | Personality and behavioural disorders due to brain disease, damage and dysfunction |
| F09 | Unspecified organic or symptomatic mental disorder |
| F102 | Mental and behavioural disorders due to use of alcohol: Dependence syndrome |
| F106 | Mental and behavioural disorders due to use of alcohol: Amnesic syndrome |
| F107 | Mental and behavioural disorders due to use of alcohol: Residual and late-onset psychotic disorder |
| F112 | Mental and behavioural disorders due to use of opioids: Dependence syndrome |
| F116 | Mental and behavioural disorders due to use of opioids: Amnesic syndrome |
| F117 | Mental and behavioural disorders due to use of opioids: Residual and late-onset psychotic disorder |
| F122 | Mental and behavioural disorders due to use of cannabinoids: Dependence syndrome |
| F126 | Mental and behavioural disorders due to use of cannabinoids: Amnesic syndrome |
| F127 | Mental and behavioural disorders due to use of cannabinoids: Residual and late-onset psychotic disorder |
| F132 | Mental and behavioural disorders due to use of sedatives or hypnotics: Dependence syndrome |
| F136 | Mental and behavioural disorders due to use of sedatives or hypnotics: Amnesic syndrome |
| F137 | Mental and behavioural disorders due to use of sedatives or hypnotics: Residual and late-onset psychotic disorder |
| F142 | Mental and behavioural disorders due to use of cocaine: Dependence syndrome |
| F146 | Mental and behavioural disorders due to use of cocaine: Amnesic syndrome |
| F147 | Mental and behavioural disorders due to use of cocaine: Residual and late-onset psychotic disorder |
| F152 | Mental and behavioural disorders due to use of other stimulants, including caffeine: Dependence syndrome |
| F156 | Mental and behavioural disorders due to use of other stimulants, including caffeine: Amnesic syndrome |
| F157 | Mental and behavioural disorders due to use of other stimulants, including caffeine: Residual and late-onset psychotic disorder |
| F162 | Mental and behavioural disorders due to use of hallucinogens: Dependence syndrome |
| F166 | Mental and behavioural disorders due to use of hallucinogens: Amnesic syndrome |
| F167 | Mental and behavioural disorders due to use of hallucinogens: Residual and late-onset psychotic disorder |
| F172 | Mental and behavioural disorders due to use of tobacco: Dependence syndrome |
| F176 | Mental and behavioural disorders due to use of tobacco: Amnesic syndrome |
| F177 | Mental and behavioural disorders due to use of tobacco: Residual and late-onset psychotic disorder |
| F182 | Mental and behavioural disorders due to use of volatile solvents: Dependence syndrome |
| F186 | Mental and behavioural disorders due to use of volatile solvents: Amnesic syndrome |
| F187 | Mental and behavioural disorders due to use of volatile solvents: Residual and late-onset psychotic disorder |
| F192 | Mental and behavioural disorders due to multiple drug use and use of other psychoactive substances: Dependence syndrome |
| F196 | Mental and behavioural disorders due to multiple drug use and use of other psychoactive substances: Amnesic syndrome |
| F197 | Mental and behavioural disorders due to multiple drug use and use of other psychoactive substances: Residual and late-onset psychotic disorder |
| F50 | Eating disorders |
| F52 | Sexual dysfunction, not caused by organic disorder or disease |
| F60 | Specific personality disorders |
| F61 | Mixed and other personality disorders |
| F62 | Enduring personality changes, not attributable to brain damage and disease |
| F63 | Habit and impulse disorders |
| F68 | Other disorders of adult personality and behaviour |
| F70 | Mild mental retardation |
| F71 | Moderate mental retardation |
| F72 | Severe mental retardation |
| F73 | Profound mental retardation |
| F78 | Other mental retardation |
| F79 | Unspecified mental retardation |
| F80 | Specific developmental disorders of speech and language |
| F81 | Specific developmental disorders of scholastic skills |
| F82 | Specific developmental disorder of motor function |
| F83 | Mixed specific developmental disorders |
| F84 | Pervasive developmental disorders |
| F88 | Other disorders of psychological development |
| F89 | Unspecified disorder of psychological development |
| F95 | Tic disorders |
| F99 | Mental disorder, not otherwise specified |
| **OTHER RESPIRATORY DISEASES** | |
| **Included ICD-10 codes and labels** | |
| B909 | Sequelae of respiratory and unspecified tuberculosis |
| E662 | Extreme obesity with alveolar hypoventilation |
| J60 | Coalworker pneumoconiosis |
| J61 | Pneumoconiosis due to asbestos and other mineral fibres |
| J62 | Pneumoconiosis due to dust containing silica |
| J63 | Pneumoconiosis due to other inorganic dusts |
| J64 | Unspecified pneumoconiosis |
| J65 | Pneumoconiosis associated with tuberculosis |
| J66 | Airway disease due to specific organic dust |
| J67 | Hypersensitivity pneumonitis due to organic dust |
| J684 | Chronic respiratory conditions due to chemicals, gases, fumes and vapours |
| J701 | Chronic and other pulmonary manifestations due to radiation |
| J703 | Chronic drug-induced interstitial lung disorders |
| J704 | Drug-induced interstitial lung disorders, unspecified |
| J84 | Other interstitial pulmonary diseases |
| J92 | Pleural plaque |
| J941 | Fibrothorax |
| J953 | Chronic pulmonary insufficiency following surgery |
| J955 | Postprocedural subglottic stenosis |
| J961 | Chronic respiratory failure |
| J98 | Other respiratory disorders |
| Q33 | Congenital malformations of lung |
| Q34 | Other congenital malformations of respiratory system |
| Z902 | Acquired absence of lung [part of] |
| Z942 | Lung transplant status |
| Z943 | Heart and lungs transplant status |
| Z963 | Presence of artificial larynx |
| **Excluded ICD-10 codes and labels** | |
| J981 | Pulmonary collapse |
| **OTHER SKIN DISEASES** | |
| **Included ICD-10 codes and labels** | |
| L13 | Other bullous disorders |
| L28 | Lichen simplex chronicus and prurigo |
| L301 | Dyshidrosis [pompholyx] |
| L43 | Lichen planus |
| L508 | Other urticaria |
| L581 | Chronic radiodermatitis |
| L85 | Other epidermal thickening |
| Q80 | Congenital ichthyosis |
| Q81 | Epidermolysis bullosa |
| Q821 | Xeroderma pigmentosum |
| Q822 | Mastocytosis |
| Q829 | Congenital malformation of skin, unspecified |
| **Excluded ICD-10 codes and labels** | |
| L432 | Lichenoid drug reaction |
| **PARKINSON AND PARKINSONISM** | |
| **Included ICD-10 codes and labels** | |
| G20 | Parkinson disease |
| G21 | Secondary parkinsonism |
| G22 | Parkinsonism in diseases classified elsewhere |
| G23 | Other degenerative diseases of basal ganglia |
| **Excluded ICD-10 codes and labels** | |
| G210 | Malignant neuroleptic syndrome |
| **PERIPHERAL NEUROPATHY** | |
| **Included ICD-10 codes and labels** | |
| B91 | Sequelae of poliomyelitis |
| G14 | Postpolio syndrome |
| G54 | Nerve root and plexus disorders |
| G55 | Nerve root and plexus compressions in diseases classified elsewhere |
| G56 | Mononeuropathies of upper limb |
| G57 | Mononeuropathies of lower limb |
| G58 | Other mononeuropathies |
| G59 | Mononeuropathy in diseases classified elsewhere |
| G60 | Hereditary and idiopathic neuropathy |
| G628 | Other specified polyneuropathies |
| G629 | Polyneuropathy, unspecified |
| G63 | Polyneuropathy in diseases classified elsewhere |
| M472 | Other spondylosis with radiculopathy |
| M531 | Cervicobrachial syndrome |
| M541 | Radiculopathy |
| **Excluded ICD-10 codes and labels** | |
| G631 | Polyneuropathy in neoplastic disease |
| **PERIPHERAL VASCULAR DISEASE** | |
| **Included ICD-10 codes and labels** | |
| I702 | Atherosclerosis of arteries of extremities |
| I73 | Other peripheral vascular diseases |
| I792 | Peripheral angiopathy in diseases classified elsewhere |
| I798 | Other disorders of arteries, arterioles and capillaries in diseases classified elsewhere |
| **Excluded ICD-10 codes and labels** | |
| I731 | Thromboangiitis obliterans |
| I738 | Other specified peripheral vascular diseases |
| **PROSTATE DISEASES** | |
| **Included ICD-10 codes and labels** | |
| N40 | Hyperplasia of prostate |
| N411 | Chronic prostatitis |
| N418 | Other inflammatory diseases of prostate |
| **SCHIZOPHRENIA AND DELUSIONAL DISEASES** | |
| **Included ICD-10 codes and labels** | |
| F20 | Schizophrenia |
| F22 | Persistent delusional disorders |
| F24 | Induced delusional disorder |
| F25 | Schizoaffective disorders |
| F28 | Other nonorganic psychotic disorders |
| **SLEEP DISORDERS** | |
| **Included ICD-10 codes and labels** | |
| F510 | Nonorganic insomnia |
| F511 | Nonorganic hypersomnia |
| F512 | Nonorganic disorder of the sleep-wake schedule |
| F513 | Sleepwalking [somnambulism] |
| G47 | Sleep disorders |
| **SOLID NEOPLASMS** | |
| **Included ICD-10 codes and labels** | |
| C | Malignant neoplasms |
| D00 | Carcinoma in situ of oral cavity, oesophagus and stomach |
| D01 | Carcinoma in situ of other and unspecified digestive organs |
| D02 | Carcinoma in situ of middle ear and respiratory system |
| D03 | Melanoma in situ |
| D04 | Carcinoma in situ of skin |
| D05 | Carcinoma in situ of breast |
| D06 | Carcinoma in situ of cervix uteri |
| D07 | Carcinoma in situ of other and unspecified genital organs |
| D09 | Carcinoma in situ of other and unspecified sites |
| D320 | Benign neoplasm: Cerebral meninges |
| D321 | Benign neoplasm: Spinal meninges |
| D329 | Benign neoplasm: Meninges, unspecified |
| D330 | Benign neoplasm: Brain, supratentorial |
| D331 | Benign neoplasm: Brain, infratentorial |
| D332 | Benign neoplasm: Brain, unspecified |
| D333 | Benign neoplasm: Cranial nerves |
| D334 | Benign neoplasm: Spinal cord |
| Q85 | Phakomatoses, not elsewhere classified |
| **Excluded ICD-10 codes and labels** | |
| C81 | Hodgkin lymphoma |
| C82 | Follicular lymphoma |
| C83 | Non-follicular lymphoma |
| C84 | Mature T/NK-cell lymphomas |
| C85 | Other and unspecified types of non-Hodgkin lymphoma |
| C86 | Other specified types of T/NK-cell lymphoma |
| C88 | Malignant immunoproliferative diseases |
| C90 | Multiple myeloma and malignant plasma cell neoplasms |
| C91 | Lymphoid leukaemia |
| C92 | Myeloid leukaemia |
| C93 | Monocytic leukaemia |
| C94 | Other leukaemias of specified cell type |
| C95 | Leukaemia of unspecified cell type |
| C96 | Other and unspecified malignant neoplasms of lymphoid, haematopoietic and related tissue |
| **THYROID DISEASES** | |
| **Included ICD-10 codes and labels** | |
| E00 | Congenital iodine-deficiency syndrome |
| E01 | Iodine-deficiency-related thyroid disorders and allied conditions |
| E02 | Subclinical iodine-deficiency hypothyroidism |
| E03 | Other hypothyroidism |
| E05 | Thyrotoxicosis [hyperthyroidism] |
| E062 | Chronic thyroiditis with transient thyrotoxicosis |
| E063 | Autoimmune thyroiditis |
| E065 | Other chronic thyroiditis |
| E07 | Other disorders of thyroid |
| E350 | Disorders of thyroid gland in diseases classified elsewhere |
| E890 | Postprocedural hypothyroidism |
| **Excluded ICD-10 codes and labels** | |
| E035 | Myxoedema coma |
| **VENOUS AND LYMPHATIC DISEASES** | |
| **Included ICD-10 codes and labels** | |
| I780 | Hereditary haemorrhagic telangiectasia |
| I83 | Varicose veins of lower extremities |
| I87 | Other disorders of veins |
| I89 | Other noninfective disorders of lymphatic vessels and lymph nodes |
| I972 | Postmastectomy lymphoedema syndrome |
| Q820 | Hereditary lymphoedema |

NOTE: When all sub-codes within a given ICD-10 code were classified as chronic, the highest possible level of aggregation of the hierarchy was included in the list (e.g. three-digit code for asthma (J45), one-digit code for malignant neoplasms (C), etc.).

**Supplemental Table 2.** UK Biobank field codes for major variables used in the present study.

| **Variable** | **Code** |
| --- | --- |
| Cardiorespiratory fitness | 20058-20060; 5983-5988, 5990, 5991 |
| Age | 21003 |
| Sex | 31 |
| Education | 6138 |
| Race | 21000 |
| Townsend deprivation index | 189 |
| Smoking Status | 20116 |
| Alcohol consumption status | 20117 |
| Physical activity | 22032 |

**Supplemental Table 3.** Hazard ratios (HRs) and 95% confidence intervals (CIs) for the association between cardiorespiratory fitness (CRF, not standardized) and multimorbidity risk.

| **CRF (not standardized)** | **No. of subjects** | **Multimorbidity risk** | | |
| --- | --- | --- | --- | --- |
|  |  | No. of cases | HR (95% CI) | P-value |
| Continuous (per 1-SD increment) | 38,348 | 15,368 | **0.97 (0.96, 0.97)** | <0.001 |
| Categorical |  |  |  |  |
| Low | 12,783 | 5,953 | 1.00 (Reference) | - |
| Moderate | 12,783 | 4,975 | **0.88 (0.84, 0.92)** | <0.001 |
| High | 12,782 | 4,440 | **0.80 (0.76, 0.84)** | <0.001 |

Abbreviation: SD, standard deviation.

Models were adjusted for age, sex, race, education, socioeconomic status, smoking status, alcohol consumption, and physical activity.

**Supplemental Table 4.** Mean time (in years) to the onset of multimorbidity, by level of cardiorespiratory fitness (CRF, not standardized).

| **CRF (not standardized)** | **No. of subjects** | **Multimorbidity onset** | | |
| --- | --- | --- | --- | --- |
|  |  | No. of cases | 50th PD (95% CI) | P-value |
| Continuous (per 1-SD increment) | 38,348 | 15,368 | **0.19 (0.14, 0.23)** | <0.001 |
| Categorical |  |  |  |  |
| Low | 12,783 | 5,953 | 0.00 (Reference) | - |
| Moderate | 12,783 | 4,975 | **0.73 (0.47, 0.99)** | <0.001 |
| High | 12,782 | 4,440 | **1.20 (0.90, 1.49)** | <0.001 |

Abbreviation: PD, percentile difference; SD, standard deviation.

Models were adjusted for age, sex, race, education, socioeconomic status, smoking status, alcohol consumption, and physical activity.

**Supplemental Table 5.** Hazard ratios (HRs) and 95% confidence intervals (CIs) for the association between cardiorespiratory fitness (CRF) and multimorbidity risk: excluding participants who were diagnosed with multimorbidity within the first 3 years of follow-up.

| **CRF** | **No. of subjects** | **Multimorbidity risk** | | |
| --- | --- | --- | --- | --- |
|  |  | No. of cases | HR (95% CI) | P-value |
| Continuous (per 1-SD increment) | 35,209 | 12,229 | **0.92 (0.90, 0.94)** | <0.001 |
| Categorical |  |  |  |  |
| Low | 11,538 | 4,374 | 1.00 (Reference) | - |
| Moderate | 11,754 | 4,112 | **0.92 (0.88, 0.96)** | 0.001 |
| High | 11,917 | 3,743 | **0.81 (0.77, 0.85)** | <0.001 |

Abbreviation: SD, standard deviation.

Models were adjusted for age, race, education, socioeconomic status, smoking status, alcohol consumption, and physical activity.

**Supplemental Table 6.** Mean time (in years) to the onset of multimorbidity, by level of cardiorespiratory fitness (CRF): excluding participants who were diagnosed with multimorbidity within the first 3 years of follow-up.

| **CRF** | **No. of subjects** | **Multimorbidity onset** | | |
| --- | --- | --- | --- | --- |
|  |  | No. of cases | 50th PD (95% CI) | P-value |
| Continuous (per 1-SD increment) | 35,209 | 12,229 | **0.35 (0.25, 0.45)** | <0.001 |
| Categorical |  |  |  |  |
| Low | 11,538 | 4,374 | 0.00 (Reference) | - |
| Moderate | 11,754 | 4,112 | **0.32 (0.08, 0.56)** | 0.005 |
| High | 11,917 | 3,743 | **0.88 (0.64, 1.12)** | <0.001 |

Abbreviation: SD, standard deviation.

Models were adjusted for age, race, education, socioeconomic status, smoking status, alcohol consumption, and physical activity.

**Supplemental Table 7.** Hazard ratios (HRs) and 95% confidence intervals (CIs) for the association between cardiorespiratory fitness (CRF) and multimorbidity risk: results from Fine-grey subdistribution hazard models.

| **CRF** | **No. of subjects** | **Multimorbidity risk** | | |
| --- | --- | --- | --- | --- |
|  |  | No. of cases | HR (95% CI) | P-value |
| Continuous (per 1-SD increment) | 38,348 | 15,368 | **0.93 (0.91, 0.94)** | <0.001 |
| Categorical |  |  |  |  |
| Low | 12,785 | 5,621 | 1.00 (Reference) | - |
| Moderate | 12,782 | 5,140 | **0.93 (0.89, 0.97)** | 0.001 |
| High | 12,781 | 4,607 | **0.82 (0.78, 0.85)** | <0.001 |

Multimorbidity is defined as the presence of at least 2 chronic diseases.

Abbreviation: SD, standard deviation.

Models were adjusted for age, race, education, socioeconomic status, smoking status, alcohol consumption, and physical activity.

**Supplemental Table 8.** Hazard ratios (HRs) and 95% confidence intervals (CIs) for the association between cardiorespiratory fitness (CRF) with multimorbidity risk: with multiple imputation of covariates

| **CRF** | **No. of subjects** | **Multimorbidity risk** | | |
| --- | --- | --- | --- | --- |
|  |  | No. of cases | HR (95% CI) | P-value |
| Continuous (per 1-SD increment) | 38,348 | 15,368 | **0.91 (0.89, 0.92)** | <0.001 |
| Categorical |  |  |  |  |
| Low | 12,785 | 5,621 | 1.00 (Reference) | - |
| Moderate | 12,782 | 5,140 | **0.89 (0.85, 0.92)** | <0.001 |
| High | 12,781 | 4,607 | **0.79 (0.75, 0.82)** | <0.001 |

Abbreviation: SD, standard deviation.

Models were adjusted for age, race, education, socioeconomic status, smoking status, alcohol consumption, and physical activity.

**Supplemental Table 9.** Mean time (in years) to the onset of multimorbidity, by level of cardiorespiratory fitness (CRF): with multiple imputation of covariates.

| **CRF** | **No. of subjects** | **Multimorbidity onset** | | |
| --- | --- | --- | --- | --- |
|  |  | No. of cases | 50th PD (95% CI) | P-value |
| Continuous (per 1-SD increment) | 38,348 | 15,368 | **0.51 (0.40, 0.63)** | <0.001 |
| Categorical |  |  |  |  |
| Low | 12,785 | 5,621 | 0.00 (Reference) | - |
| Moderate | 12,782 | 5,140 | **0.48 (0.23, 0.72)** | <0.001 |
| High | 12,781 | 4,607 | **1.29 (1.02, 1.55)** | <0.001 |

Abbreviation: SD, standard deviation.

Models were adjusted for age, race, education, socioeconomic status, smoking status, alcohol consumption, and physical activity.

**Supplemental Table 10.** Standardized betas (β) and 95% confidence intervals (CIs) for the association between cardiorespiratory fitness (CRF) and number of chronic diseases accumulated over the 15-year follow-up: with multiple imputation of covariates.

| **CRF** | **All Chronic disease** | |  | **Metabolic disease** | |  | **Cardiovascular disease** | |  | **Neuropsychiatric disease** | |
| --- | --- | --- | --- | --- | --- | --- | --- | --- | --- | --- | --- |
|  | β (95% CI) | P-value |  | β (95% CI) | P-value |  | β (95% CI) | P-value |  | β (95% CI) | P-value |
| Continuous (per 1-SD increment) × time | **-0.018 (-0.021, -0.016)** | <0.001 |  | **-0.008 (-0.009, -0.007)** | <0.001 |  | **-0.001 (-0.002, -0.001)** | <0.001 |  | **-0.001 (-0.001, -0.000)** | <0.001 |
| Categorical × time |  |  |  |  |  |  |  |  |  |  |  |
| Low | 0.000 (reference) | - |  | 0.000 (reference) | - |  | 0.000 (reference) | - |  | 0.000 (reference) | - |
| Moderate | **-0.023 (-0.030, -0.018)** | <0.001 |  | **-0.012 (-0.014, -0.011)** | <0.001 |  | **-0.002 (-0.004, -0.001)** | 0.002 |  | -0.000 (-0.001, 0.001) | 0.561 |
| High | **-0.044 (-0.050, -0.038)** | <0.001 |  | **-0.019 (-0.021, -0.017)** | <0.001 |  | **-0.003 (-0.004, -0.001)** | <0.001 |  | **-0.002 (-0.003, -0.001)** | 0.001 |

Abbreviation: SD, standard deviation.

Models were adjusted for age, race, education, socioeconomic status, smoking status, alcohol consumption, and physical activity.
